# Building photoswitchable 3,4'-AMPB peptides: Probing chemical ligation methods with reducible azobenzene thioesters

**DOI:** 10.3762/bjoc.8.101

**Published:** 2012-06-18

**Authors:** Gehad Zeyat, Karola Rück-Braun

**Affiliations:** 1 Institut für Chemie, Technische Universität Berlin, Strasse des 17. Juni 135, 10623 Berlin, Germany

**Keywords:** acyl transfer auxiliary, azobenzenes, ligation, molecular switches, peptides, redox chemistry

## Abstract

Photoswitchable peptides were synthesized by using cysteine- and auxiliary-based native chemical ligation reactions. For this purpose, the two regioisomeric azobenzene building blocks 3,4'-AMPB thioester **1b** and 4,4'-AMPB thioester **2b** were employed in the ligation reactions. While 4,4'-AMPB requires the 4,5,6-trimethoxy-2-mercaptobenzyl auxiliary to minimize reduction of the diazene unit, 3,4'-AMPB can be used in combination with the 4,5,6-trimethoxy-2-mercaptobenzyl auxiliary as well as the *N*^α^-2-mercaptoethyl auxiliary. Thus, 3,4'-AMPB derivatives/peptides proved to be significantly less prone to reduction by aliphatic and aromatic thiols than were the 4,4'-AMPB compounds.

## Introduction

**Figure 1:**
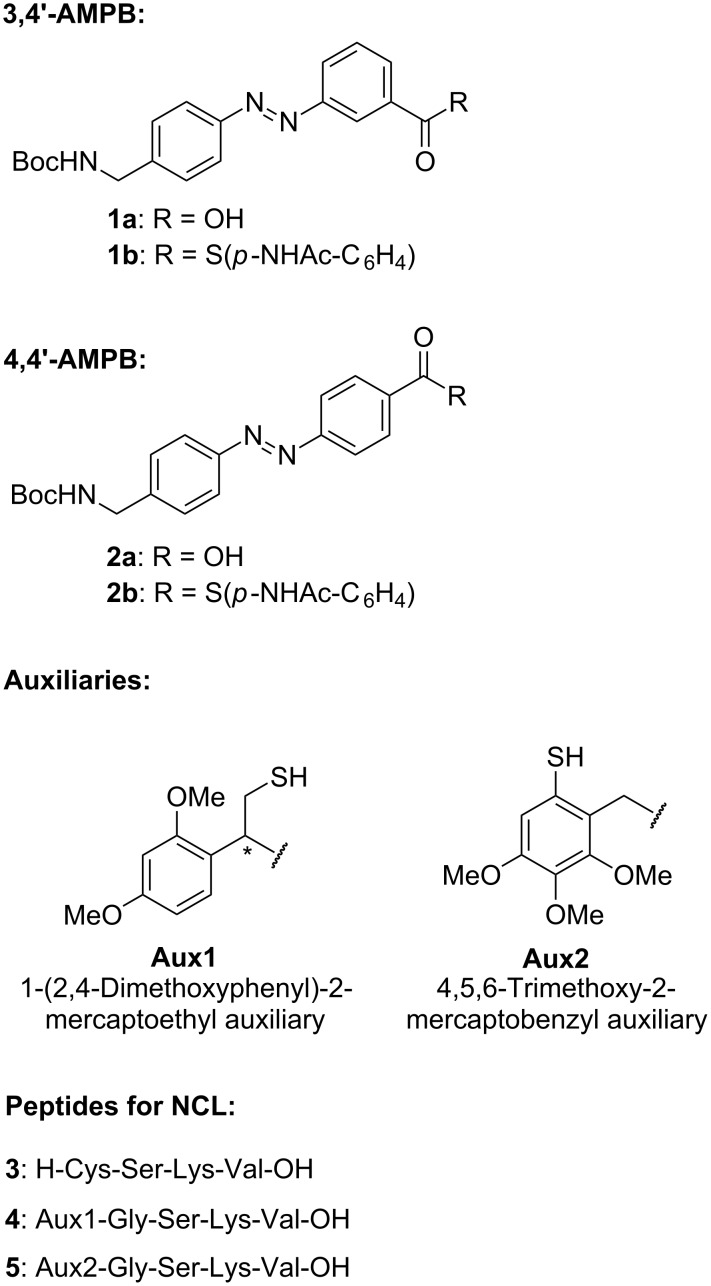
Structures of azobenzene thioesters, *N*^α^-ligation auxiliaries and peptides for the application in ligation studies. AMPB = (aminomethylphenylazo)benzoic acid.

Optical switches not only offer the advantage to elucidate, but also to control biological processes with high spatial and temporal resolution by using light, either in vitro or in vivo [1]. In this context, azobenzenes remain a privileged class of photoswitches, and typical applications based on light-triggered reversible conformational control of cyclic, helical or beta-hairpin peptides have been intensively reviewed [2–3]. Current research in this field focuses on advanced methods and designs for the synthesis of complex azopeptides and azoproteins for ambitious biophysical studies, and also for intracellular applications. In this respect, an increased and predictable stability of azobenzene building blocks under reducing conditions seems to be a prerequisite in light of synthetic challenges, but also when considering the reducing intracellular environment. We recently reported on the synthesis, properties and applications of a series of novel azobenzene ω-amino acids, with a preference for meta-substitution patterns. Our purpose was amongst other things (i) to increase flexibility, and (ii) to suppress resonance effects in order to enhance the stability of the diazene unit [4–5]. Since methods based on native chemical ligation (NCL), e.g., NCL with *N*^α^-acyl transfer auxiliaries, have gained considerable interest in the past two decades [6], we reasoned that a comparative ligation study with the Boc-protected azobenzene ω-amino acids Boc-3,4'-AMPB **1a** and Boc-4,4'-AMPB **2a** may result in an in-depth analysis of the redox-stability of these building blocks under the reducing conditions of thiol-based ligation methods. Generally, native chemical ligation allows the coupling of two unprotected peptides in neutral aqueous solution: a C-terminal thioester peptide and either an N-terminal cysteine peptide or *N*^α^-auxiliary-capped peptides. However, for elucidating the complex redox chemistry of the two azobenzene building blocks under the reducing conditions of ligation methods, we solely applied the Boc-protected azobenzene ω-amino acid thioesters **1b** and **2b** instead of a C-terminal thioester peptide. We explored the conventional cysteine-based NCL with Cys-peptide **3**, and also screened the application of the TFA-cleavable 4,5,6-trimethoxy-2-mercaptobenzyl (Tmb) and 1-(2,4-dimethoxyphenyl)-2-mercaptoethyl auxiliaries by using peptides **4** and **5** (Figure 1) [6–8], in order to circumvent the need for the presence of a cysteine at the ligation site.

**Scheme 1:**
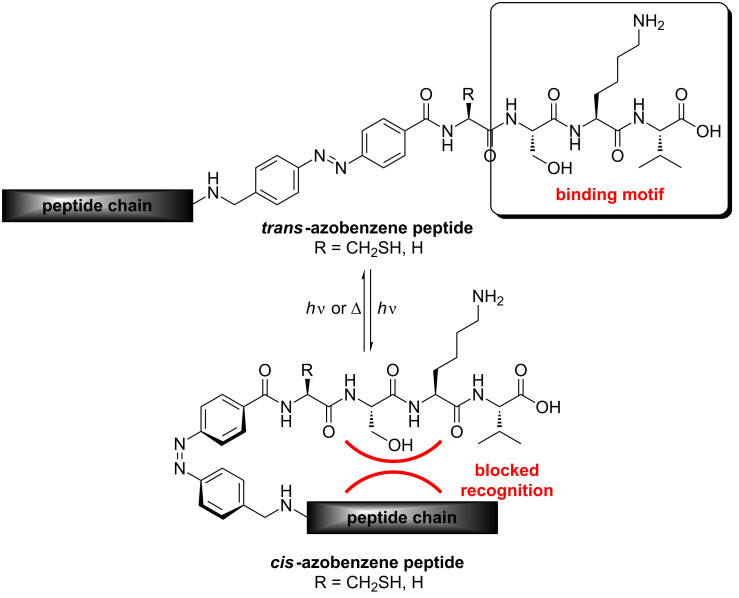
Structural differences between the *trans*- and the *cis*-state of azopeptides with a SKV PDZ binding motif.

The peptide motif in peptides **3**, **4** and **5** is based on a specific binding sequence for PDZ recognition [9]. Numerous scaffolding proteins contain multiple PDZ domains for their interaction with PDZ-binding motifs at the C-terminus of transmembrane channels and receptors or other intracellular signaling proteins. Class 1 interactions involve a (S/T)-X-(V/I/L)-COOH sequence motif, and examples for these interactions include the proteins PSD-95, Fas or NHERF 1 [10]. Fas (APO-1/CD95), for example, is a cell surface receptor, belonging to the tumor-necrosis-factor receptor superfamily, which induces apoptosis. Fas-associated phosphatase-1 (FAP-1) is a Fas binding protein, which interacts with 12 to 15 of the C-terminal amino acids of the Fas receptor; however, the necessary and sufficient region for binding consists of the three C-terminal amino acids (SLV) [11–13]. PDZ-domain-containing proteins are superb examples of allosteric systems built up by semirigid domains able to interact by means of flexible regions, and therefore they seem to be ideally suited for biophysical function studies with photoswitchable ligands. For instance, Fas-associated studies in certain cells using the tripeptide SLV suggest that this small peptide alone can induce apoptosis [14–15]. Related peptides containing class I C-terminal sequence motifs, e.g., SKV, are also derived from viral origins [16]. The latter specific binding motif is associated with H5N1 influenza infections. We reasoned that the attachment of a photoswitch next to SXV PDZ-binding motifs would be a powerful strategy for exploring the function of these PDZ-binding peptide ligands in vitro and in vivo. For the synthetic ligation studies presented herein we had to place the ligation junction next to the third amino acid serine of the SKV binding motif. In the case of the conventional NCL, we used cysteine at the ligation site, and for the auxiliary mediated ligations, we decided to introduce auxiliary-glycine-conjugates at the N-terminus, due to the steric limitations of this type of ligation reaction [6–8]. After ligation, the SKV binding motif in the photoswitchable azopeptides should only be accessible in the dark *trans*-azo state (Scheme 1), whereas light switching to the *cis*-azo state should result in unfavorable interactions between the N-terminus, which follows behind the azo-switch, and the side chains that are critical for PDZ recognition (as exemplified in Scheme 1 for the 4,4'-substituted azobenzene system). However, the design presented herein, in combination with an appropriate ligation method, seems also to be applicable to the synthesis and evaluation of photoswitchable protein analogues, for example receptors with the photoswitch placed in the direct neighborhood of the specific C-terminal binding motif.

## Results and Discussion

**Figure 2:**
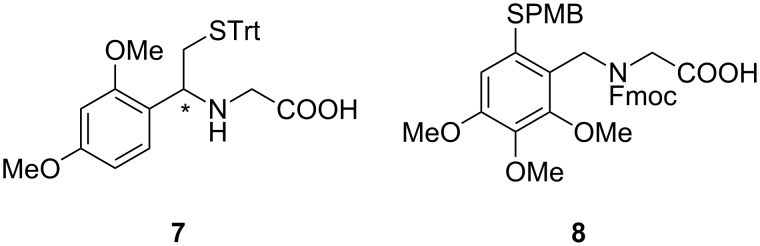
Structure of the glycine-linked auxiliary conjugates **7** and **8**.

The azobenzene ω-amino-acid thioesters **1b** and **2b** were prepared by following and applying literature procedures (Supporting Information File 1) [4,17]. The syntheses of the auxiliary-linked glycine conjugates **7** and **8** were accomplished by using literature protocols developed by Dawson, Offer and MacMillan [7–8] (Figure 2).

**Scheme 2:**
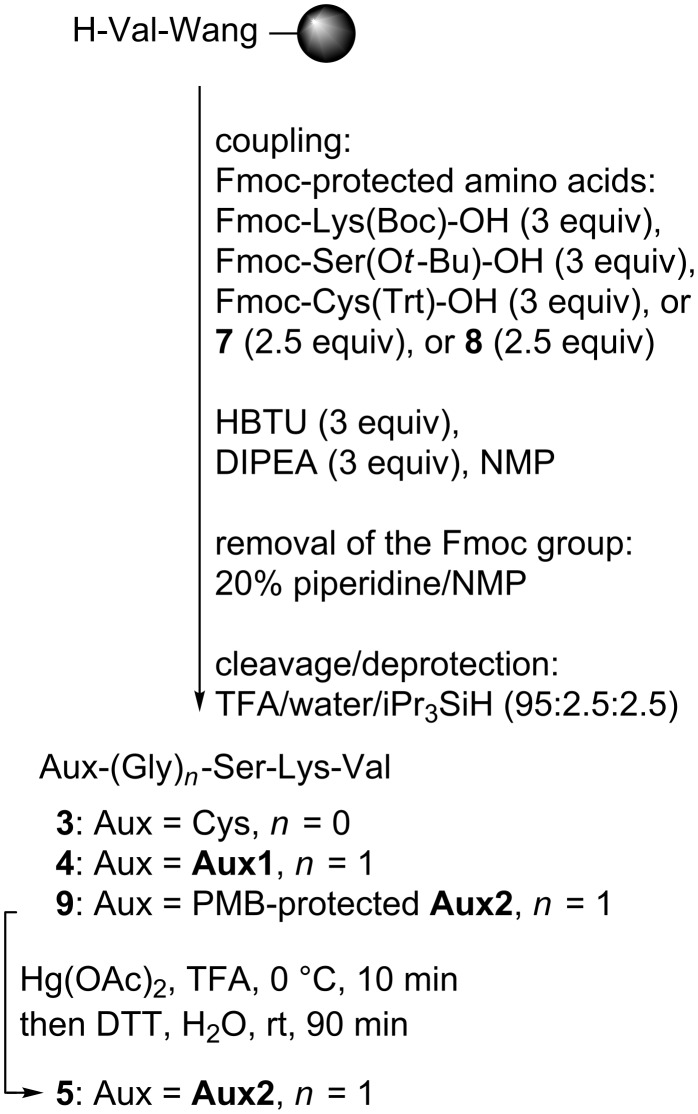
Solid-phase synthesis of the ligation-mediating peptides **3**–**5**.

The Cys-peptide **3** and the *N*^α^-auxiliary peptides **4** and **5** were assembled on Wang resin by manual Fmoc-based peptide synthesis using standard amino-acid building blocks (Scheme 2) [4]. Commercially available preloaded Wang resin was used, and HBTU was applied as the coupling reagent in the presence of DIPEA in NMP. Removal of the temporary Fmoc-protecting group was achieved by using 20% piperidine in NMP. Cleavage from the resin and removal of the permanent *tert*-butyl and trityl protecting groups was carried out with a mixture of trifluoroacetic acid, water and triisopropylsilane. The peptides **3** and **4** were purified by preparative RP-HPLC and isolated in 86% and 56% yield, respectively. Peptide **9** with a protected thiol moiety was treated with Hg(OAc)_2_ in TFA, followed by DTT according to the literature [8], and the deprotected peptide **5** was purified by preparative RP-HPLC and isolated in 64% yield.

All attempts to ligate azobenzene ω-amino acid thioester **1b** in phosphate buffer in the presence of TCEP·HCl and guanidinium hydrochloride failed, because of the poor solubility of the azobenzene thioester in the aqueous buffer as well as in buffer/NMP mixtures. Therefore, a ligation protocol of Danishefsky et al. was applied using DMF as the solvent [18]. The ligations were realized by reaction of the appropriate peptide **3**, **4** or **5** with an excess of the azobenzene thioester (1.9 equiv), and TCEP·HCl (2.75 equiv) in the presence of Na_2_HPO_4_ (5.5 equiv, pH 7.3) at room temperature, and the reaction courses were monitored by analytical RP-HPLC (Supporting Information File 1). The ligation reactions were conducted under normal lighting conditions, and therefore small amounts of the *cis*-azobenzene forms of the thioesters **1b**/**2b** and of the ligation products **10**–**15** were detected during HPLC monitoring (Supporting Information File 1). The results of all ligation courses prior to purification by chromatography are summarized in Table 1.

**Table 1:** Cysteine- and auxiliary-based ligation courses with 3,4'-AMPB thioester **1b** and 4,4'-AMPB thioester **2b**.^a^

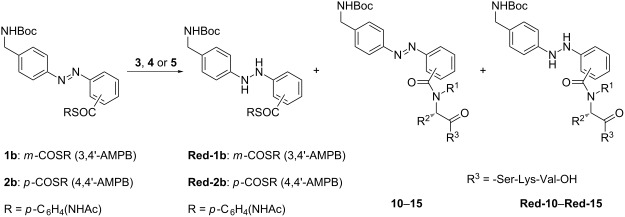								

No.	Thioester	Peptide	Red-**1b**/**2b**^b^ [%]	Product	R^1^	R^2^	Ratio of product/red-product^c^	Yield^d^ [%]

1	**1b**	**3**	–	**10**	H	CH_2_SH	69:31	62
2	**1b**	**4**	9	**11**	Aux1	H	97:3	43
3	**1b**	**5**	–	**12**	Aux2	H	100:0	38
4	**2b**	**3**	4	**13**	H	CH_2_SH	52:48	44
5	**2b**	**4**	14 (12)^e^	**14**	Aux1	H	18:82 (21:79)^e^	43
6	**2b**	**5**	2	**15**	Aux2	H	90:10	45

^a^Reaction conditions: 1.9 equiv of azobenzene thioester **1b** or **2b**, TCEP·HCl (2.75 equiv), Na_2_HPO_4_ (5.5 equiv), DMF, rt. Peptides **3–5** were employed as the symmetric disulfides and were reduced in situ by TCEP. ^b^Conversion of **1b** or **2b** to the hydrazines **Red-1b** or **Red-2b** related to the unreacted excess of **1b** or **2b** in the ligation mixture during HPLC-monitoring after the appropriate reaction time; – : **Red-1b** was not detected. ^c^HPLC-based ratio determined by HPLC-monitoring after work-up and lyophilization of the crude ligation mixture. ^d^Isolated yield after preparative RP-HPLC. ^e^Reinvestigated ratio given in brackets.

In ligation reactions employing the N-terminal Cys-peptide **3**, nearly complete conversion was determined after five hours, but stirring was nevertheless continued overnight (Table 1, entries 1 and 4). For peptide **5** containing the 4,5,6-trimethoxy-2-mercaptobenzyl auxiliary, complete consumption of the starting material was detected after 23–24 h (Table 1, entries 3 and 6). In ligation reactions employing the *N*^α^-2-mercaptoethyl auxiliary peptide **4** reaction times were doubled for complete turnover (Table 1, entries 2 and 5). These results are in accordance with a chemoselective bimolecular thioester exchange prior to the S- to N-acyl transfer through a five-membered transition state for Cys-peptide **3**, a six-membered transition state for 4,5,6-trimethoxy-2-mercaptobenzyl auxiliary peptide **5**, and a sterically more demanding five-membered transition state for racemic *N*^α^-2-mercaptoethyl auxiliary peptide **4** [6].

During the reaction courses employing Cys-peptide **3** and the 4,5,6-trimethoxy-2-mercaptobenzyl auxiliary peptide **5**, only reduction of the 4,4'-AMPB thioester **2b** to the corresponding hydrazine compound **Red-2b** was observed, in 2–4% yield (Table 1). When using the *N*^α^-2-mercaptoethyl auxiliary peptide **4**, reduction of both azobenzene thioesters was detected: Formation of 9% **Red-1b** was observed for the 3,4'-AMPB thioester **1b**, and of 14% **Red-2b** for 4,4'-AMPB thioester **2b**. Obviously, the excess of the azobenzene thioester prohibits reduction of the ligation products. Additional experiments showed that the yields of the ligation products could be improved by dilution of the reaction solutions with water after complete conversion of the peptide starting materials, followed by extraction of the aqueous phase with diethylether and ethyl acetate to remove 4-acetamidothiophenol (Aatp), derived from the thioesters, as well as the excess of the azobenzene thioesters **1b** and **2b**. After lyophilization, analysis of the crude products by RP-HPLC indicated the formation of varying amounts of reduced ligation peptides in five out of six cases. Severe reduction was observed in ligation reactions with Cys-peptide **3** and 3,4'-AMPB thioester **1b**, with a ratio of 69:31 peptide **10**/peptide **Red-10**, as well as 4,4'-AMPB thioester **2b**, with a ratio of 52:48 peptide **13**/peptide **Red-13** (Table 1, entries 1 and 4). Moreover, the *N*^α^-2-mercaptoethyl auxiliary peptide **4** in combination with 4,4'-AMPB thioester **2b** gave peptide **14**/peptide **Red14** in an 18:82 ratio (Table 1, entry 5). However, reoxidation of the hydrazine ligation peptides **Red-10**, **Red-13** and **Red-14** to the azobenzene ligation peptides **10**, **13** and **14** was observed after purification by preparative RP-HPLC, and is obviously initiated by oxygen from the air. Finally, the pure ligated azobenzene peptides **10**, **13** and **14** were isolated in yields of 62%, 44% and 43%, respectively. When 3,4'-AMPB thioester **1b** and the *N*^α^-2-mercaptoethyl auxiliary peptide **4** were used, the desired peptide **11** and its hydrazine analogue **Red-11** were detected in a ratio of 97:3 by analytical RP-HPLC (Table 1, entry 2), and peptide **11** was isolated in 43% yield after preparative RP-HPLC. In light of the reactivity of the *N*^α^-2-mercaptoethyl auxiliary, reduction of the azobenzene ligation peptides derived from peptide **4** can obviously only be avoided by using 3,4'-AMPB thioester **1b**, due to the lower reactivity of the diazene unit in this thioester owing to the meta-substitution pattern.

The ligation reaction of 3,4'-AMPB thioester **1b** with the 4,5,6-trimethoxy-2-mercaptobenzyl auxiliary peptide **5** furnished ligation peptide **12** in 38% yield (Table 1, entry 3). In this case, no hydrazine species **Red-12** were detected at all (Table 1, entry 3). In addition, the ligation reaction of 4,5,6-trimethoxy-2-mercaptobenzyl auxiliary peptide **5** and 4,4'-AMPB thioester **2b** was also successfully accomplished, furnishing a ratio of 90:10 for **15**/**Red-15**, and the pure ligation product **15** was isolated in 45% yield (Table 1, entry 6). Thus, the 4,5,6-trimethoxy-2-mercaptobenzyl auxiliary applied in peptide **5** seems to be of general use for both azobenzene systems **1b** and **2b**, with a manageable tendency toward reduction in the case of the 4,4'-AMPB thioester **2b**.

**Scheme 3:**
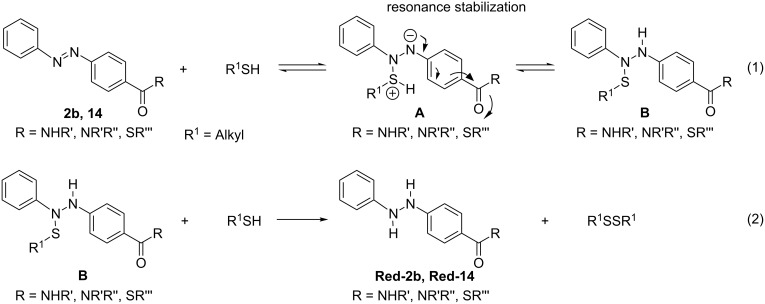
Reduction of the diazene unit of 4,4'-AMPB thioesters and peptides during aliphatic thiol-based *N*^α^-auxiliary ligation strategies.

The reduction sensitivity observed for the 4,4'-AMPB-derived thioester **2b** and its ligation peptides, may be rationalized by the following considerations [19–20]: The initial addition of a thiol to an electron-poor diazene double bond is a reversible reaction, furnishing a zwitterionic adduct intermediate **A** en route to an *N*-sulfenohydrazodiarene **B**, with a rather labile N–S bond (Scheme 3, reaction 1). Aliphatic thiols readily undergo adduct formation towards the *N*-sulfenohydrazodiarene intermediates **B** because of their higher nucleophilicity. Accordingly, we observe a higher tendency toward reduction when applying the aliphatic *N*^α^-2-mercaptoethyl auxiliary in comparison to the aromatic 4,5,6-trimethoxy-2-mercaptobenzyl auxiliary. Furthermore, only 4,4'-substituted azobenzene systems, e.g., **2b** and peptide **14**, furnish resonance-stabilized zwitterionic intermediates **A**. Finally, reduction is completed by a nucleophilic attack of the second aliphatic thiol molecule at the *N*-sulfenohydrazodiarene **B**, yielding the symmetric disulfide and the respective hydrazine thioester or hydrazine peptide (Scheme 3, reaction 2). The reduction of diazene units by thiols has been intensively studied in the past for electron-poor azobenzenes or azodicarboxylates, en route to symmetrically and asymmetrically substituted disulfides [21–23].

**Scheme 4:**
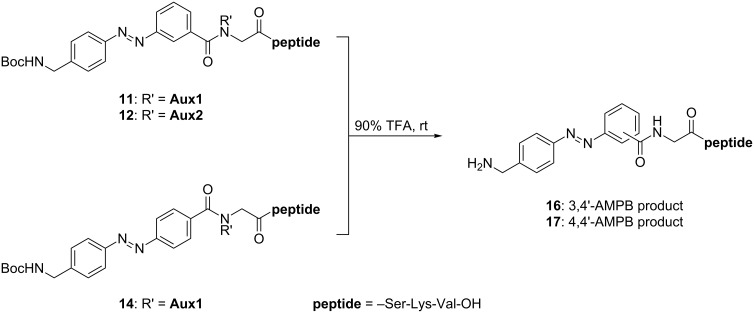
Synthesis of the azopeptides **16**/**17** by final TFA cleavage of the Boc-protecting groups, and of the auxiliaries **Aux1** and **Aux2** in the ligation peptides **11**, **12** and **14**.

The final cleavage of the Boc-protecting group, the *N*^α^-2-mercaptoethyl auxiliary and the 4,5,6-trimethoxy-2-mercaptobenzyl auxiliary in the 3,4'-AMPB ligation peptides **12** and **14** was successfully achieved with 90% TFA under standard conditions furnishing peptide **16** in 94% and 89% yield, respectively, after purification by preparative RP-HPLC (Scheme 4). Similarly, the Boc-protected 4,4'-AMPB ligation peptide **14** derived from the *N*^α^-2-mercaptoethyl auxiliary peptide **4** was deprotected and purified yielding peptide **17** in 81% yield.

## Conclusion

In conclusion, we have demonstrated the high redox stability of the Boc-protected 3,4'-AMPB thioester **1b** when applying *N*^α^-auxiliary ligation methods en route to photoswitchable peptides containing the C-terminal PDZ binding motif SKV. The positioning of the carbonyl group relative to the diazene unit in the 3,4'-AMPB-derived thioester **1b** and its peptides predictably decreases the reactivity towards unwanted redox side-reactions upon application of aliphatic as well as aromatic thiols in *N*^α^-auxiliary ligation strategies using peptides **4** and **5**. In stark contrast, the Boc-protected 4,4'-AMPB thioester reproducibly furnished peptide **15** only in combination with the 4,5,6-trimethoxy-2-mercaptobenzyl auxiliary peptide **5** during the ligation course and work-up, whereas the *N*^α^-2-mercaptoethyl auxiliary peptide **4** preferentially gave the hydrazine ligation peptide **Red-14** besides the azobenzene ligation peptide **14**. Our future efforts directed towards the synthesis of photoswitchable peptides will focus on water-soluble 3,4'-AMPB building blocks being applicable in native chemical ligation methods in aqueous media.

## Supporting Information

File 1 Experimental procedures, characterization data and copies of spectra.

## References

[1] Wachtveitl J, Zumbusch A (2011). ChemBioChem.

[2] Renner C, Moroder L (2006). ChemBioChem.

[3] Beharry A A, Wong L, Tropepe V, Woolley G A (2011). Angew Chem, Int Ed.

[4] Rück-Braun K, Kempa S, Priewisch B, Richter A, Seedorff S, Wallach L (2009). Synthesis.

[5] Hoppmann C, Schmieder P, Domaing P, Vogelreiter G, Eichhorst J, Wiesner B, Morano I, Rück-Braun K, Beyermann M (2011). Angew Chem, Int Ed.

[6] Offer J (2010). Biopolymers.

[7] Offer J, Boddy C N C, Dawson P E (2002). J Am Chem Soc.

[8] Macmillan D, Anderson D W (2004). Org Lett.

[9] Harris B Z, Lim W A (2001). J Cell Sci.

[10] Walma T, Spronk C A E M, Tessari M, Aelen J, Schepens J, Hendriks W, Vuister G W (2002). J Mol Biol.

[11] Yanagisawa J, Takahashi M, Kanki H, Yano-Yanagisawa H, Tazunoki T, Sawa E, Nishitoba T, Kamishohara M, Kobayashi E, Kataoka S (1997). J Biol Chem.

[12] Freiss G, Chalbos D (2011). Anti-Cancer Agents Med Chem.

[13] Subbaiah V K, Kranjec C, Thomas M, Banks L (2011). Biochem J.

[14] Ungefroren H, Kruse M-L, Trauzold A, Roeschmann S, Roeder C, Arlt A, Henne-Bruns D, Kalthoff H (2001). J Cell Sci.

[15] Huang W, Zhu C, Wang H, Horvath E, Eklund E A (2008). J Biol Chem.

[16] Javier R T, Rice A P (2011). J Virol.

[17] Priewisch B, Rück-Braun K (2005). J Org Chem.

[18] Wu B, Chen J, Warren J D, Chen G, Hua Z, Danishefsky S J (2006). Angew Chem, Int Ed.

[19] Linke K-H, Brandt W, Göhausen H J (1973). Chem Ber.

[20] Boulègue C, Löweneck M, Renner C, Moroder L (2007). ChemBioChem.

[21] Mukaiyama T, Takahashi K (1968). Tetrahedron Lett.

[22] Wünsch E, Moroder L, Romani S (1982). Hoppe-Seyler’s Z Physiol Chem.

[23] Kosower E M, Kanety-Londner H (1976). J Am Chem Soc.

