# Supplementary material for: Building photoswitchable 3,4'-AMPB peptides: Probing chemical ligation methods with reducible azobenzene thioesters
Source: Beilstein J Org Chem. 2012 Jun 18;8:890–6. doi: 10.3762/bjoc.8.101 (PMC3388879; doi:10.3762/bjoc.8.101)
Supplement: File 1 — Experimental procedures, characterization data and copies of spectra. [file Beilstein_J_Org_Chem-08-890-s001.pdf]

Supporting Information

for

Building photoswitchable 3,4'-AMPB peptides: Probing chemical ligation methods with reducible azobenzene thioesters

Gehad Zeyat and Karola Rück-Braun*

Address: Institut für Chemie, Technische Universität Berlin, Strasse des 17. Juni 135, 10623 Berlin, Germany

Email: Karola Rück-Braun - karola.rueck-braun@tu-berlin.de

* Corresponding author

Experimental procedures, characterization data and copies of spectra.

Table of contents

Materials and methods S3

Synthesis of azobenzene building blocks S4

3-((4-(*tert*-Butoxycarbonylaminomethyl)phenyl)diazenyl)benzoic acid (Boc-3,4'-AMPB) (**1a**) S4

4-((4-(*tert*-Butoxycarbonylaminomethyl)phenyl)diazenyl)benzoic acid (Boc-4,4'-AMPB) (**2a**) S5

Synthesis of azobenzene thioesters **1b** and **2b** S6

Solid phase peptide synthesis S7

Synthesis of H-Cys-Ser-Lys-Val-OH (**3**) S7

Auxiliary-capped peptides **4** and **9** S8

PMB-cleavage of crude peptide **9** and purification of peptide **5** S10

Native chemical ligation using peptide **3** S11

Ligation of azobenzene thioester **1b** with peptide **3**: Peptide **10** S11

LC–MS and UV–vis data for the ligation course of peptide **10** S12

LC–MS and UV–vis data on the formation of reduced peptide **Red-10** S14

Ligation of azobenzene thioester **2b** with peptide **3**: Peptide **13** S14

LC–MS and UV–vis data for the ligation course of peptide **13** S15

LC–MS and UV–vis data on the formation of reduced ligation peptide **Red-13** S17

Cysteine-free native chemical ligation S18

Ligation of azobenzene thioester **1b** with peptide **4**: Peptide **11** S18

LC–MS and UV–vis data for the ligation course of peptide **11** S19

LC–MS and UV–vis data on the formation of reduced peptide **Red-11** S21

Ligation of azobenzene thioester **1b** with peptide **5**: Peptide **12** S21

LC–MS and UV–vis data for the ligation course of peptide **12** S22

Ligation of azobenzene thioester **2b** with peptide **4**: Peptide **14** S24

LC–MS and UV–vis data for the ligation course of peptide **14** S24

LC–MS and UV–vis data on the formation of reduced peptide **Red-14** S26

Ligation of azobenzene thioester **2b** with peptide **5**: Peptide **15** S27

LC–MS and UV–vis data for the ligation course of peptide **15** S28

LC–MS and UV–vis data on the formation of reduced peptide **Red-15** S29

TFA-mediated deprotection and cleavage of the auxiliaries S30

Synthesis of peptide **16** S30

Synthesis of peptide **17** S31

Analytical data of synthesized peptides S32

NMR spectra of compounds **1a**, **2a**, **1b**, **2b**, **3**–**5** and **10**, **13**, **16** and **17** S33

References S48

Materials and methods

All starting materials and reagents were purchased at the highest available purity and were used without further purification. Thin-layer chromatography (silica, Merck 60 F_{254} plates) was visualized by UV light, ninhydrine or acidic phosphomolybdic acid/cerium(IV) sulfate stain. Preparative flash chromatography was performed on ICN Biomedicals GmbH silica gel 60 (32–63 µm, 60 Å). Melting points were recorded on a Büchi melting point apparatus M-560 using open capillary tubes, and remain uncorrected. Infrared spectra were recorded as ATR (attenuated total reflectance) on a Nicolet FT-IR 750 spectrometer and are reported as wavenumbers in cm^{-1}.

NMR spectra were recorded on a Bruker AM 400 spectrometer or on a Bruker DRX 500 spectrometer at 298 K. The frequencies used for ^{1}H NMR and ^{13}C NMR spectra are mentioned for the particular substances. Chemical shifts are given as dimensionless δ values and coupling constants in Hz. Solvents are mentioned for the particular substances; their residual signals were used as an internal standard. The number of protons was determined by integration of the corresponding signals.

Electrospray-ionization mass spectra (ESIMS) were recorded in positive mode on a Bruker Esquire 2000 with an ESI voltage of 4 kV and HRMS–ESI spectra on a Thermo Fisher Scientific LTQ Orbitrap XL with an ESI voltage of 5 kV.

Analytical RP-HPLC was performed on an HP Agilent 1100 system with a diode array detector and online monitoring at 210, 254 and 440 nm. An RP-C18 column Phenomenex Luna (5.0 µm, 250 × 4.60 mm) with a flow rate of 1.0 mL/min was used. Preparative RP-HPLC was performed on an Agilent HPLC 1260 infinity system using a Zorbax C18 column (21.2 × 150 mm, 5.0 µm, Agilent) with a multiple wavelength detector at a flow rate of 20 mL/min. In all cases the employed binary mobile phase systems consisted either of acetonitrile (A) and water (B) or of 0.1% (v/v) TFA in acetonitrile (C) and 0.1% (v/v) TFA in water (D). The following elution gradients were applied: (Conditions A): Isocratic elution with 5% A and 95% B for five minutes, followed by a linear gradient to 40% A and 60% B in 10 minutes; (Conditions B): Isocratic elution with 5% A and 95% B for five minutes, followed by a linear gradient to 35% A and 65% B in 10 minutes; (Conditions C): Isocratic

elution with 5% A and 95% B for five minutes, followed by a linear gradient to 20% A and 80% B in 4 minutes, followed by another linear gradient to 50% A and 50% B in 15 minutes; (Conditions D): Isocratic elution with 5% A and 95% B for five minutes, followed by a linear gradient to 30% A and 70% B in 6 minutes, followed by another linear gradient to 50% A and 50% B in 10 minutes; (Conditions E): Isocratic elution with 5% A and 95% B for five minutes, followed by a linear gradient to 35% A and 65% B in 20 minutes; (Conditions F): Linear gradient of 40% A and 60% B to 80% A and 20% B in 20 minutes; (Conditions G): Isocratic elution with 5% C and 95% D for five minutes, followed by a linear gradient to 100% A in 20 minutes; (Conditions H): Isocratic elution with 5% C and 95% D for five minutes, followed by a linear gradient to 100% A in 40 minutes; (Conditions J): Linear gradient of 40% C and 60% D to 80% C and 20% D in 20 minutes. The applied elution gradient is noted for each peptide.

Synthesis of azobenzene building blocks

3-((4-(*tert*-Butoxycarbonylaminomethyl)phenyl)diazenyl)benzoic acid (Boc-3,4'-AMPB) (1a)

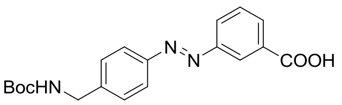

3-Nitrosobenzoic acid [1] (3.17 g, 21.0 mmol, 2 equiv) was dissolved in glacial AcOH/DMSO (1:1, 400 mL), and *tert*-butyl 4-aminobenzylcarbamate [1] (2.33 g, 10.5 mmol, 1 equiv) was added over a period of 30 min in small portions. The reaction mixture was stirred at rt for 20 h. Water (200 mL) was added and the resulting precipitate was separated by filtration using a suction filter, washed with water (100 mL) and recrystallized from methanol to afford compound **1a** (2.36 g, 6.6 mmol, 63%) as an orange solid. Mp 217 °C; R_f 0.49 (ethyl acetate/hexane 2:1); ^{1}H NMR (d_6-DMSO, 400 MHz): δ = 8.37 (t, ^4J = 1.6 Hz, 1H), 8.15–8.10 (m, 2H), 7.91 (d, ^3J = 8.2 Hz, 2H), 7.53 (t, ^3J = 7.9 Hz, 1H), 7.46 (d, ^3J = 8.4 Hz, 2H), 4.23 (d, ^3J = 8.4 Hz, 2H), 1.41 (s, 9H) ppm; ^{13}C NMR (d_6-DMSO, 100.6 MHz): δ = 166.8, 155.9, 152.0, 150.8, 144.6, 132.2, 131.8, 130.1, 128.0, 127.5, 122.9, 122.2, 78.1, 43.3, 28.3 ppm; IR (ATR):

ῡ = 3346, 1695, 1682, 1516, 1249, 1169, 680 cm^{-1}; MS (ESI) *m*/*z* (%) = 378 (80) [M + Na]$^+$, 356 (50) [M + H]$^+$, 220 (90), 178 (100); HRMS (ESI) calcd. for [$C_{19}H_{22}N_3O_4$]$^+$: 356.1605; found: 356.1606.

4-((4-(*tert*-Butoxycarbonylaminomethyl)phenyl)diazenyl)benzoic acid (Boc-4,4'-AMPB) (2a)

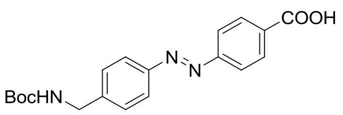

To a solution of 4-((4-((9*H*-fluoren-9-yl)methoxycarbonyl-aminomethyl)phenyl)diazenyl)benzoic acid [1] (800 mg, 1.68 mmol, 1.0 equiv) in DMF (17 mL) potassium fluoride (1.02 g, 17.6 mmol, 10.5 equiv), triethylamine (526 mg, 0.74 mL, 5.19 mmol, 3.1 equiv) and Boc_2O (731 mg, 3.35 mmol, 2.0 equiv) were added [2], and the suspension was stirred at rt. After 36 h, the reaction mixture was diluted with ethyl acetate (20 mL) and washed with water (3 × 10 mL), citric acid (10% w/v aq. sol., 3 × 10 mL), $NaHCO_3$ (5% w/v aq. sol., 3 × 10 mL) and brine (3 × 10 mL). The organic layer was dried (Na_2SO_4) and concentrated under reduced pressure. Silica gel chromatography (ethyl acetate → ethyl acetate/acetic acid 100:1) afforded compound **2a** (493 mg, 1.39 mmol, 83%) as an orange solid. Mp 396 °C (decomposition); R_f 0.44 (ethyl acetate/hexane 2:1); ^{1}H NMR (d_6-DMSO, 400 MHz): δ = 8.14 (d, ^3J = 8.5 Hz, 2H), 7.95 (d, ^3J = 8.6 Hz, 2H), 7.90 (d, ^3J = 8.3 Hz, 2H), 7.51 (t, ^3J = 6.2 Hz, 1H), 7.47 (d, ^3J = 8.4 Hz, 2H), 4.23 (d, ^3J = 6.1 Hz, 2H), 1.41 (s, 9H) ppm; ^{13}C NMR (d_6-DMSO, 100.6 MHz): δ = 166.8, 155.9, 154.4, 150.9, 144.9, 132.9, 130.7, 128.0, 123.0, 122.6, 78.1, 43.3, 28.3 ppm; IR (ATR): ῡ = 3340, 2961, 2925, 2848, 1681, 1603, 1506, 1427, 1305, 1288, 1263, 1249, 1169, 1052, 946, 867, 840, 776, 691 cm^{-1}; MS (ESI) *m*/*z* (%) = 378 (100) [M + Na]$^+$; HRMS (ESI) calcd. for [$C_{19}H_{21}N_3O_4Na$]$^+$: 378.1424; found: 378.1426.

Synthesis of azobenzene thioesters 1b and 2b

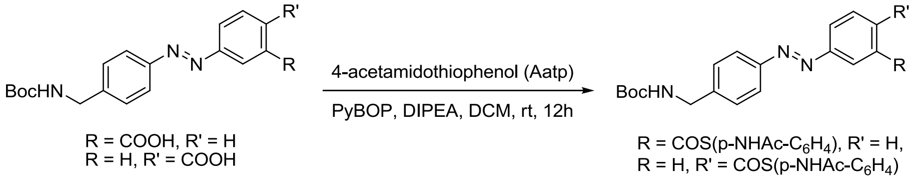

General procedure [3]

To a suspension of azobenzene **1a** or **2a** (1.0 equiv) in DCM (c = 28 mmol/L), PyBOP (1.3 equiv) and 4-acetamidothiophenol (1.3 equiv) were added at rt. Upon addition of DIPEA (1.3 equiv) the reaction mixture turned into a clear solution and was stirred for 12 h. The reaction mixture was then washed with water and brine, dried (Na_2SO_4) and concentrated under reduced pressure. The crude product was recrystallized from ethanol to give the desired thioester.

S-4-Acetamidophenyl 3-((4-(*tert*-butoxycarbonylaminomethyl)phenyl)diazenyl)benzothioate (1b)

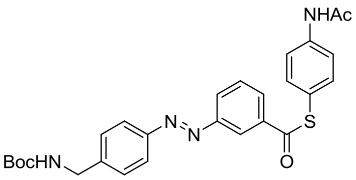

The reaction was carried out with 400 mg of Boc-3,4'-AMPB **1a** (1.13 mmol) as described in the general procedure to give 474 mg (0.94 mmol, 83%) of the photochromic thioester **1b** as an orange solid. Mp 188 °C; R_f 0.56 (ethyl acetate/hexane 2:1); ^{1}H NMR (d_6-DMSO, 400 MHz): δ = 10.21 (s, 1H), 8.35 (t, ^4J = 1.8 Hz, 1H), 8.23–8.20 (m, 1H), 8.15–8.12 (m, 1H), 7.92 (d, ^3J = 8.3 Hz, 2H), 7.81 (t, ^3J = 7.9 Hz, 1H), 7.73 (d, ^3J = 8.7 Hz, 2H), 7.53 (t, ^3J = 6.2 Hz, 1H), 7.48–7.46 (m, 4H), 4.24 (d, ^3J = 6.1 Hz, 2H), 2.09 (s, 3H), 1.41 (s, 9H) ppm; ^{13}C NMR (d_6-DMSO, 100.6 MHz): δ = 189.4, 168.7, 155.9, 152.1, 150.7, 144.7, 140.9, 137.1, 135.7, 130.7, 129.4, 128.2, 127.9, 127.3, 122.9, 119.9, 119.6, 119.3, 78.0, 43.3, 28.2, 24.1 ppm. IR (ATR): ῦ = 3333, 3306, 1684, 1666, 1592, 1517, 1396, 1249, 1170, 826, 686 cm^{-1}; MS (ESI) m/z (%) = 527 (48) [M + Na]$^+$, 223 (32) [M + H]$^+$, 167 (100), 106 (64); HRMS (ESI) calcd. for $[C_{27}H_{29}N_4O_4S]^+$: 505.1904; found: 505.1904.

S-4-Acetamidophenyl 4-((4-(*tert*-butoxycarbonylaminomethyl)phenyl)diazenyl)benzothioate (2b)

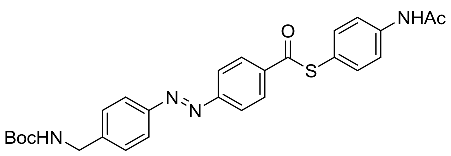

The reaction was carried out with 100 mg of Boc-4,4'-AMPB **2a** (0.28 mmol) as described in the general procedure to give 116 mg (0.23 mmol, 82%) of the photochromic thioester **2b** as an orange solid. Mp 213 °C; R_f 0.71 (ethyl acetate/hexane 2:1); ^{1}H NMR (d_6-DMSO, 400 MHz): δ = 10.21 (s, 1H), 8.18 (d, ^3J = 8.5 Hz, 2H), 8.03 (d, ^3J = 8.5 Hz, 2H), 7.93 (d, ^3J = 8.3 Hz, 2H), 7.73 (d, ^3J = 8.5 Hz, 2H), 7.54 (t, ^3J = 6.1 Hz, 1H), 7.49–7.46 (m, 4H), 4.24 (d, ^3J = 6.0 Hz, 2H), 2.09 (s, 3H), 1.41 (s, 9H) ppm; ^{13}C NMR (d_6-DMSO, 100.6 MHz): δ = 189.2, 168.8, 155.9, 154.9, 150.9, 145.1, 140.9, 137.3, 135.7, 128.5, 127.9, 123.1, 123.0, 119.6, 119.3, 78.0, 43.2, 28.2, 24.1 ppm; IR (ATR) ῦ = 3313, 2978, 1713, 1688, 1665, 1651, 1538, 1398, 1203, 905, 849, 839, 822 cm^{-1}; MS (ESI) *m*/*z* (%) = 527 (100) [M + Na]$^+$; HRMS (ESI) calcd. for [$C_{27}H_{28}N_4O_4SNa$]$^+$: 527.1723; found: 527.1723.

Solid phase peptide synthesis

Synthesis of H-Cys-Ser-Lys-Val-OH (3)

Peptide **3** was synthesized manually on a 0.1 mmol scale by standard protocols using the Fmoc/*tert*-Butyl strategy [4]. The appropriate amount of the commercially available preloaded Wang resin Fmoc-Val-Wang (0.51 mmol/g, 196 mg, 0.1 mmol) was swollen for 20 min in DCM (5 mL) prior to use.

Manual solid phase peptide synthesis – General procedure

The *N*-Fmoc-protected amino acid (0.3 mmol, 3 equiv), HBTU (114 mg, 0.3 mmol, 3 equiv), DIPEA (50 µL, 0.3 mmol, 3 equiv) and NMP (6 mL) were added to the resin and the mixture was vortexed for 30 min. After each coupling step, the resin was washed with NMP (3 × 4 mL) and DCM (3 × 4 mL).

The Fmoc group was removed by first treating the resin with a 20% solution of piperidine in NMP (5 mL) for 5 min and then by retreatment of the resin with the same solution (5 mL) for 10 min. After each deprotection step, the resin was thoroughly washed with NMP (3 × 4 mL) and DCM (3 × 4 mL).

Cleavage of the peptide from the resin was accomplished by treatment with TFA/H_2O/iPr_3SiH (190:5:5, 6 mL) for 2.5 h. The resin was removed by filtration and washed with TFA/H_2O/iPr_3SiH

(190:5:5, 2 × 3 mL). The cleavage mixture was concentrated in vacuo by azeotropic distillation with hexane (three times) and afterwards precipitated in cold diethyl ether. Then the precipitate was dissolved in water and lyophilized.

Purification of the crude peptide 3

The crude peptide (70 mg) was purified by RP-HPLC on an Agilent preparative HPLC using a Zorbax C18 column (21.2 × 150 mm, 5 µm, Agilent) at a flow rate of 20 mL/min with spectrometric monitoring at λ = 210 nm, using HPLC conditions B to give 57 mg (43 µmol, 86%) of peptide **3** as the disulfide (colorless solid).

^{1}H NMR (DMSO-d_6, 500 MHz): δ = 12.68 (br s, Val1-COO*H*), 8.69 (d, ^3J = 7.5 Hz, 1H, Ser3-NH), 8.11 (d, ^3J = 8.0 Hz, 1H, Lys2-NH), 7.97 (d, ^3J = 8.4 Hz, 1H, Val1-NH), 7.78 (br s, 3H, Lys2-NH_3^+), 5.15 (br s, 1H, Ser3-OH), 4.42–4.33 (m, 2H, Ser3-C$H_{(\alpha)}$, Lys2-C$H_{(\alpha)}$), 4.12–4.09 (m, 1H, Val1-C$H_{(\alpha)}$), 4.07 (t, ^3J = 5.3 Hz, 1H, Cys4-C$H_{(\alpha)}$), 3.65–3.58 (m, 2H, Ser3-C$H_{2(\beta)}$), 3.01–2.87 (m, 2H, Cys4-C$H_{2(\beta)}$), 2.74 (br. s, 2H, Lys2-C$H_{2(\varepsilon)}$), 2.06–2.02 (m, 1H, Val1-C$H_{(\beta)}$), 1.72–1.51 (m, 4H, Lys2-C$H_{(\beta1,2)}$, Lys2-C$H_{(\delta1,2)}$)), 1.36–1.29 (m, 2H, Lys2-C$H_{(\gamma)}$), 0.87 (dd, ^3J = 6.7 Hz, ^4J = 1.7 Hz, 6H, Val1-C$H_{(\gamma)}$) ppm.

Auxiliary-capped peptides 4 and 9

Peptides **4** and **9** were synthesized manually on a 0.2 mmol scale by standard protocols using the Fmoc/*tert*-Butyl strategy. The appropriate amount of the commercially available preloaded Wang resin (0.51 mmol/g, 392 mg, 0.2 mmol) was swollen for 20 min in DCM (6 mL) prior to use.

Manual solid phase peptide synthesis – General procedure

For the synthesis of auxiliary-capped peptide **4**, the *N*-Fmoc-protected amino acid (0.6 mmol, 3 equiv) or the glycine-linked auxiliary conjugate **7** [5] (0.5 mmol, 2.5 equiv, 257 mg), HBTU (228 mg, 0.6 mmol, 3 equiv), DIPEA (99 µL, 0.6 mmol, 3 equiv) and NMP (12 mL) were added to the resin and the mixture was vortexed for 30 min.

The same procedure was carried out for the synthesis of auxiliary-capped peptide **9**, but instead of **7**, the *N*-Fmoc-protected glycine-bound auxiliary **8** [5] (0.5 mmol, 2.5 equiv, 378 mg) was used in the third coupling step. After each coupling step, the resin was washed with NMP (3 × 8 mL) and DCM (3 × 8 mL).

The Fmoc group was removed by treatment of the resin with a 20% solution of piperidine in NMP (8 mL) for 5 min, followed by retreatment with the same solution (5 mL) for 10 min. After each deprotection step, the resin was thoroughly washed with NMP (3 × 8 mL) and DCM (3 × 8 mL).

Cleavage of the peptide from the resin was accomplished by treatment with TFA/H_2O/iPr$_3$SiH (190:5:5, 12 mL) for 2.5 h. The resin was removed by filtration and washed with TFA/H_2O/iPr$_3$SiH (190:5:5, 2 × 6 mL). The cleavage mixture was concentrated in vacuo by azeotropic distillation with hexane (three times) and the peptide was precipitated in cold diethyl ether. Then the precipitate was dissolved in water and lyophilized.

Purification of the crude peptide 4

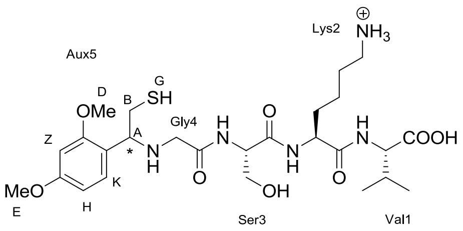

The crude peptide (128 mg) was purified by RP-HPLC on an Agilent preparative HPLC using a Zorbax C18 column (21.2 × 150 mm, 5 µm, Agilent) at a flow rate of 20 mL/min with spectrometric monitoring at λ = 210 nm, using HPLC conditions B to give 78 mg (56 µmol, 56%) of a diastereomeric mixture of peptide **4** as the disulfide (colorless solid).

^{1}H NMR (DMSO-d_6, 400 MHz): δ = 8.11–8.09 (m, 1H, Gly4-NH), 7.90–7.87 (m, 1H, Ser3-NH), 7.71 (br. s, 3H, Lys2-NH_3^+), 7.26 (d, ^3J = 8.2 Hz, 1H, Aux5-C$H_{(K)}$), 6.57 (s, 2H, Aux5-C$H_{(H)}$, Aux5-C$H_{(Z)}$), 5.02 (br. s, 1H, Ser3-OH), 4.39–4.29 (m, 3H, Ser3-C$H_{(\alpha)}$, Lys2-C$H_{(\alpha)}$, Aux5-C$H_{(A)}$), 4.11–4.08 (m, 1H, Val1-C$H_{(\alpha)}$), 3.78–3.70 (m, 6H, Aux5-OC$H_{3(D,E)}$), 3.56–3.49 (m, 3H, Ser3-C$H_{2(\beta)}$, Gly4-C$H_{2(\alpha1,2)}$), 2.74 (br. s, 1H, Lys2-C$H_{2(\varepsilon)}$), 2.06–2.01 (m, 1H, Val1-C$H_{(\beta)}$), 1.71–1.68 (m, 4H, Lys2-C$H_{(\beta1,2)}$, Lys2-C$H_{(\delta1,2)}$)), 1.31–1.24 (m, 2H, Lys2-C$H_{(\gamma)}$), 0.86 (d, ^3J = 6.6 Hz, 6H, Val1-C$H_{(\gamma)}$) ppm.

PMB-cleavage of crude peptide 9 and purification of peptide 5

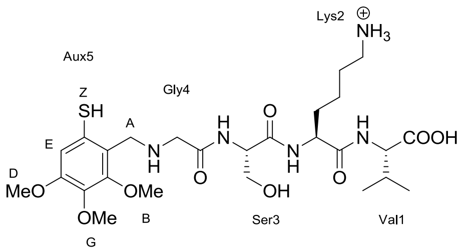

The crude peptide **9** (163 mg) was dissolved in ice-cold TFA (10 mL) at 0 °C, and $Hg(OAc)_2$ (0.51 mmol, 164 mg) was added to the solution. The reaction mixture was gently shaken under N_2 atmosphere at 0 °C for 10 min and was subsequently diluted with water (90 mL). Solid DTT (1.08 mmol, 166 mg) was added and the reaction mixture was shaken for 90 min at rt. The white precipitate was removed by centrifugation and the remaining solution was lyophilized. The crude product was purified by RP-HPLC on an Agilent preparative HPLC using a Zorbax C18 column (21.2 × 150 mm, 5 µm, Agilent) at a flow rate of 20 mL/min with spectrometric monitoring at λ = 210 nm, under HPLC conditions A to give 92 mg (64 µmol, 64%) of peptide **5** as the disulfide (colorless solid).

^{1}H NMR (d_6-DMSO, 500 MHz): δ = 8.81 (br. s, 1H, Gly4-NH), 8.59 (d, ^3J = 7.1 Hz, 1H, Ser3-NH), 8.17 (d, ^3J = 7.1 Hz, 1H, Lys2-NH), 7.96 (d, ^3J = 7.1 Hz, 1H, Val1-NH), 7.76 (br. s, 3H, Lys2-NH_3^+), 6.85 (s, 1H, TMB5-C$H_{(E)}$), 4.47–4.43 (m, 1H, Ser3-C$H_{(\alpha)}$), 4.34–4.30 (m, 1H, Lys2-C$H_{(\alpha)}$), 4.12–4.09 (m, 1H, Val1-C$H_{(\alpha)}$), 3.89–3.74 (m, 11H, TMB5-OC$H_{3(B,G,D)}$, TMB5-C$H_{2(A)}$), 3.63–3.50 (m, 4H, Ser3-C$H_{2(\beta)}$, Gly4-C$H_{2(\alpha1,2)}$), 2.73 (br. s, 1H, Lys2-C$H_{2(\varepsilon)}$), 2.07–2.01 (m, 1H, Val1-C$H_{(\beta)}$), 1.74–1.50 (m, 4H, Lys2-C$H_{(\beta1,2)}$, Lys2-C$H_{(\delta1,2)}$)), 1.36–1.23 (m, 2H, Lys2-C$H_{(\gamma)}$), 0.87 (d, ^3J = 6.6 Hz, 6H, Val1-C$H_{(\gamma)}$) ppm.

Native chemical ligation using peptide 3

Typical procedure [6]

To a solution of the disulfide of peptide **3** (1 equiv) in DMF (0.5 mmol/L, HPLC-grade), TCEP × HCl (5.5 equiv), Na_2HPO_4 (11 equiv) and the photochromic thioester (3.8 equiv, neat) were added and the pH of the reaction mixture was detected and adjusted with additional amounts of Na_2HPO_4 to pH 7.3 after sonication. The mixture was then stirred at rt under an atmosphere of nitrogen.

After the HPLC analysis of the ligation mixture showed complete conversion of peptide **3**, the ligation mixture was diluted with water at 0 °C to obtain a 1:1 mixture of DMF/water and washed three times with ice-cold diethyl ether and three times with ice-cold ethyl acetate.

Ligation of azobenzene thioester 1b with peptide 3: Peptide 10

10 and **Red-10**:

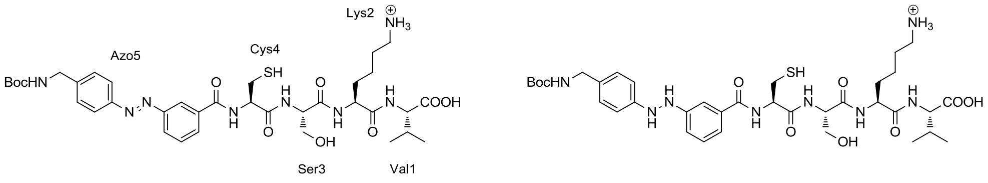

The ligation was carried out with 30 mg of the disulfide of peptide **3** (22.6 µmol, 1 equiv) in 45 mL of DMF. TCEP × HCl (36 mg, 124 µmol, 5.5 equiv), Na_2HPO_4 (36 mg, 254 µmol, 11 equiv) and the azobenzene thioester **1b** (43 mg, 85.9 µmol, 3.8 equiv) were added, and the reaction was worked up as described in the typical procedure after complete consumption of peptide **3** (reaction time: 25 h). After lyophilization of the aqueous layer, the crude product was purified by RP-HPLC on an Agilent preparative HPLC using a Zorbax C18 column (21.2 × 150 mm, 5 µm, Agilent) at a flow rate of 20 mL/min with spectrometric monitoring at λ = 210 nm and λ = 450 nm using HPLC conditions C to give the disulfide of the photochromic peptide **10** (2.8 µmol, 5 mg, 12%) as a light-yellow solid in addition to the disulfide of the reduced peptide **Red-10** (11.2 µmol, 19.9 mg, 50%). Peptide **Red-10** was obtained in a colorless solution and turned upon exposure to air into the desired peptide product **10**. Therefore, an overall yield of 62% (24.9 mg, 14.0 µmol) was achieved for the photochromic peptide **10** (disulfide).

^{1}H NMR (DMSO-d_6, 500 MHz): δ = 8.98 (br. s, 1H, Ser3-NH), 8.38–8.34 (m, 1H, Lys2-NH), 8.15–8.11 (m, 2H, 2 × Azo5-CH), 8.03–7.94 (m, 2H, Val1-NH, 1 × Azo5-CH), 7.90–7.81 (m, 3H, 3 × Azo5-CH), 7.68–7.59 (m, 2H, Cys4-NH, 1 × Azo5-CH), 7.52–7.44 (m, 2H, 1 × Azo5-CH, Azo5-N*H*), 4.87 (br. s, 1H, Cys4-C$H_{(\alpha)}$), 4.34–4.28 (m, 2H, Ser3-C$H_{(\alpha)}$, Lys2-C$H_{(\alpha)}$), 4.23–4.16 (m, 2H, Azo5-CH_2), 4.06–4.03 (m, 1H, Val1-C$H_{(\alpha)}$), 3.63–3.58 (m, 2H, Ser3-C$H_{2(\beta)}$), 3.07–3.04 (m, 1H, Cys4-C$H_{2(\beta)}$), 2.73–2.69 (m, 2H, Lys2-C$H_{2(\varepsilon)}$), 2.02 (br. s, 1H, Val1-C$H_{(\beta)}$), 1.75–1.52 (m, 4H, Lys2-C$H_{(\beta 1,2)}$, Lys2-C$H_{(\delta 1,2)}$), 1.41–1.23 (m, 11H, Azo5-Boc(CH_3)$_3$, Lys2-C$H_{2(\gamma)}$), 0.85–0.84 (m, 6H, Val1-C$H_{3(\gamma)}$) ppm.

LC–MS and UV–vis data for the ligation course of peptide 10

The HPLC analyses of the ligation time course were performed after filtration of the ligation mixture to remove solid Na_2HPO_4 using HPLC conditions H with spectrometric monitoring at λ = 210 nm (Figure S1, left) and λ = 450 nm (Figure S1, right).

The formation of the *cis*-product $\mathbf{10}_{\boldsymbol{cis}}$ (signal c, t_R = 29.8 min) and the *trans*-product $\mathbf{10}_{\boldsymbol{trans}}$ (signal d, t_R = 31.1 min) was detected in considerable amounts after 2 h (ESIMS: *m/z* ($\mathbf{10}_{\boldsymbol{cis}}$) = 773.4; *m/z* ($\mathbf{10}_{\boldsymbol{trans}}$) = 773.5 ($[M + H]^+$, calculated: 773.4), Figure S2). The UV–vis spectra of the ligation products $\mathbf{10}_{\boldsymbol{cis}}$ ($\lambda_{max,1}$ = 234 nm; $\lambda_{max,2}$ = 430 nm; shoulder at λ = 290 nm, Figure S3, left) and $\mathbf{10}_{\boldsymbol{trans}}$ ($\lambda_{max,1}$ = 232 nm; $\lambda_{max,2}$ = 326 nm; $\lambda_{max,3}$ = 437 nm, Figure S3, right) show typical characteristics for peptides containing azoaromatics of the azobenzene-type, according to the classification of Rau, showing two clearly separated absorption maxima for the low lying n–π* transition and for the π–π* transition [7].

Furthermore, an increasing signal at t_R = 29.2 min (signal b, Figure S1) was detected, which belongs to the disulfide of the thiol leaving group 4-acetamidothiophenol (Aatp, structure shown in Figure S2, ESIMS: *m/z* = 355.1 ($[M + Na]^+$, calculated: 355.1)).

Although the cysteinyl peptide **3** was nearly consumed completely after 5 h, the ligation mixture was stirred overnight for a total of 25 h.

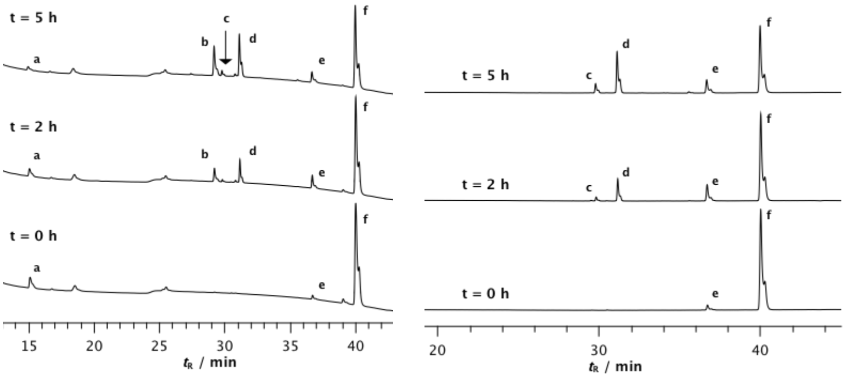

Figure S1: HPLC traces of the reaction course for the synthesis of peptide **10**, detection at 210 nm (left) and 450 nm (right): (a) cysteinyl peptide **3**; (b) thiol leaving group Aatp as disulfide; (c) *cis*-ligation product $\mathbf{10}_{\boldsymbol{cis}}$; (d) *trans*-ligation product $\mathbf{10}_{\boldsymbol{trans}}$; (e) *cis*-azobenzene thioester $\mathbf{1b}_{\boldsymbol{cis}}$; (f) *trans*-azobenzene thioester $\mathbf{1b}_{\boldsymbol{trans}}$.

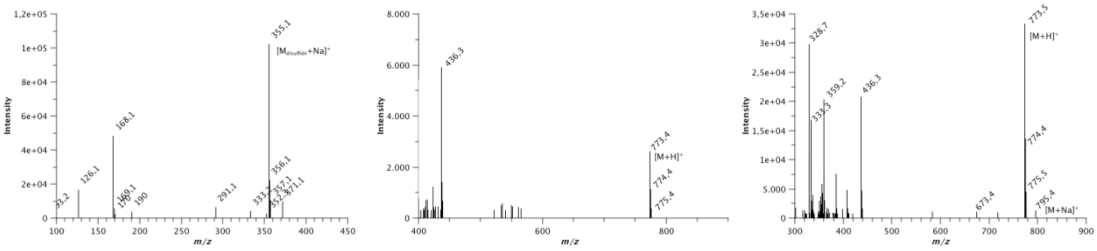

Figure S2: ESIMS spectra of the HPLC signals: b (Aatp as disulfide, left), c ($\mathbf{10}_{\boldsymbol{cis}}$, middle) and d ($\mathbf{10}_{\mathbf{trans}}$, right).

Disulfide of the thiol leaving group Aatp:

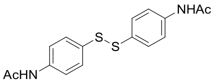

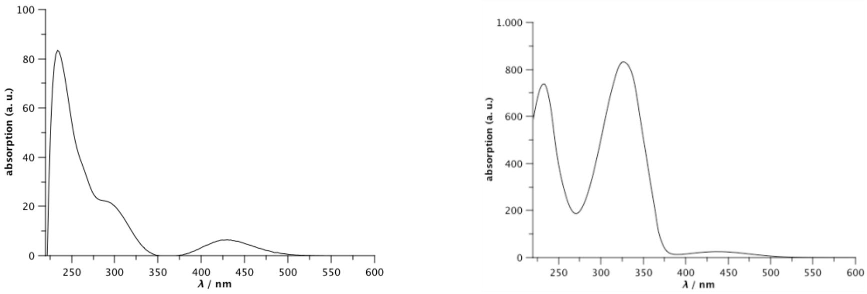

Figure S3: UV–vis spectra: *cis*-ligation product $\mathbf{10}_{\boldsymbol{cis}}$ (left), and *trans*-ligation product $\mathbf{10}_{\boldsymbol{trans}}$ (right).

LC–MS and UV–vis data on the formation of reduced peptide Red-10

The LC–MS analysis of the crude product after extractive workup and lyophilization revealed a mixture of the desired ligation product **10** in its *cis*- and *trans*-form and the reduced diaryl hydrazine peptide **Red-10** (Figure S4). Due to the disrupted π-delocalization in this hydrazine compound compared to the corresponding azobenzene peptide **10**, the UV–vis spectrum lacks the long-wavelength absorption band at λ = 410–550 nm (Figure S4, right). Upon integration of the signals detected at λ = 210 nm, an HPLC-based ratio of the compounds $\mathbf{10}_{\mathit{cis/trans}}$/**Red-10** of 69:31 is determined in the crude product.

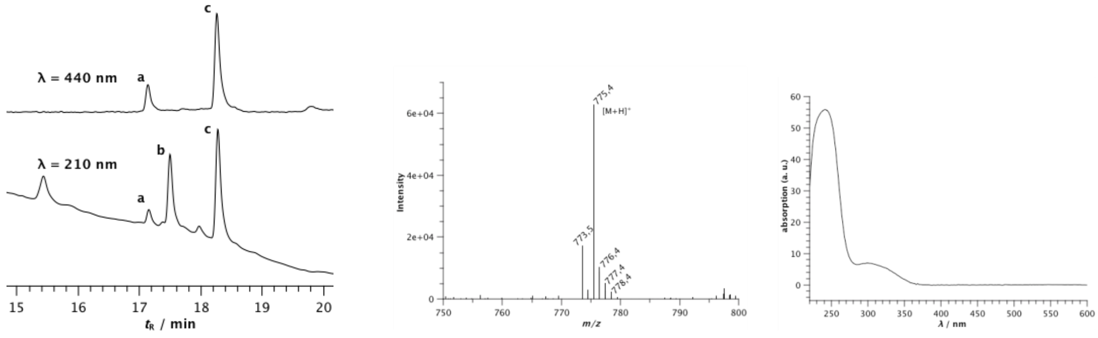

Figure S4: *Left*: HPLC traces of the ligation mixture after extractive workup and lyophilization (HPLC conditions G): (a) *cis*-ligation product $\mathbf{10}_{\mathit{cis}}$; (b) reduced ligation product **Red-10**; (c) *trans*-ligation product $\mathbf{10}_{\mathit{trans}}$. *Middle*: ESIMS spectrum of HPLC signal b. *Right*: UV–vis data of HPLC signal b.

Ligation of azobenzene thioester 2b with peptide 3: Peptide 13

13 and **Red-13**:

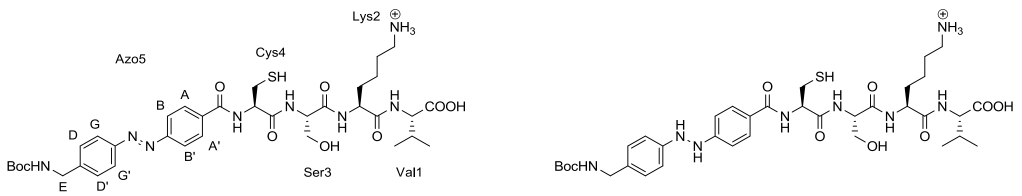

The ligation was carried out with 8.0 mg of the disulfide of peptide **3** (6.0 µmol, 1 equiv) in 12 mL of DMF. TCEP × HCl (9.5 mg, 33.2 µmol, 5.5 equiv), Na_2HPO_4 (9.6 mg, 67.6 µmol, 11 equiv) and the azobenzene thioester **2b** (11.6 mg, 22.9 µmol, 3.8 equiv) were added, and the reaction was worked up as described in the typical procedure after complete consumption of peptide **3** (reaction time: 21 h). After lyophilization of the aqueous layer, the crude product was purified by RP-HPLC on an Agilent

preparative HPLC using a Zorbax C18 column (21.2 × 150 mm, 5 µm, Agilent), at a flow rate of 20 mL/min with spectrometric monitoring at λ = 210 nm and λ = 440 nm using HPLC conditions D, to give the disulfide of the photochromic peptide **13** (2.9 mg, 1.6 µmol, 27%) as a light-yellow solid in addition to the disulfide of the reduced peptide **Red-13** (1.8 mg, 1.0 µmol, 17%). Peptide **Red-13** was obtained in a colorless solution and turned upon exposure to air into the desired product **13**. Therefore, an overall yield of 44% (4.7 mg, 2.6 µmol) was achieved for the yellow peptide **13** (disulfide).

^{1}H NMR (DMSO-d_6, 400 MHz): δ = 8.78 (d, ^3J = 7.8 Hz, 1H, Ser3-NH), 8.23 (d, ^3J = 7.6 Hz, 1H, Lys2-NH), 8.12 (d, ^3J = 8.5 Hz, 2H, Azo5-CH$_{(G,G')}$), 8.07–8.01 (m, 1H, Val1-NH), 7.97 (d, ^3J = 8.6 Hz, 2H, Azo5-CH$_{(B,B')}$), 7.90 (d, ^3J = 8.3 Hz, 2H, Azo5-CH$_{(A,A')}$), 7.88–7.86 (m, 1H, Cys4-NH), 7.52 (t, ^3J = 6.0 Hz, 1H, Azo5-N*H*), 7.47 (d, ^3J = 8.4 Hz, 2H, Azo5-CH$_{(D,D')}$), 4.65–4.64 (m, 1H, Cys4-C$H_{(\alpha)}$), 4.36–4.31 (m, 2H, Ser3-C$H_{(\alpha)}$, Lys2-C$H_{(\alpha)}$), 4.23 (d, ^3J = 6.0 Hz, 2H, Azo5-C$H_{2(E)}$), 4.09–4.07 (m, 1H, Val1-C$H_{(\alpha)}$), 3.67–3.56 (m, 2H, Ser3-C$H_{2(\beta)}$), 3.00–2.87 (m, 2H, Cys4-C$H_{2(\beta)}$), 2.76–2.72 (m, 2H, Lys2-C$H_{2(\varepsilon)}$), 2.05–2.01 (m, 1H, Val1-C$H_{(\beta)}$), 1.72–1.53 (m, 4H, Lys2-C$H_{(\beta1,2)}$, Lys2-C$H_{(\delta1,2)}$), 1.41–1.23 (m, 11H, Azo5-Boc(CH_3)$_3$, Lys2-C$H_{2(\gamma)}$), 0.87 (d, ^3J = 6.7 Hz, 6H, Val1-C$H_{3(\gamma)}$).

LC–MS and UV–vis data for the ligation course of peptide 13

The HPLC analyses of the ligation time course were performed after filtration of the ligation mixture to remove solid Na_2HPO_4 using HPLC conditions G with spectrometric monitoring at λ = 210 nm (Figure S5, left) and λ = 440 nm (Figure S5, right).

The formation of the *cis*-product **13$_{cis}$** (signal c, t_R = 17.1 min) and the *trans*-product **13$_{trans}$** (signal d, t_R = 18.2 min) was detected after 1 h (ESIMS: *m/z* (**13$_{cis}$**) = 773.3; *m/z* (**13$_{trans}$**) = 773.4 ([M + H]$^+$, calculated: 773.4), Figure S6). The UV–vis spectra of the ligation products **13$_{cis}$** (shoulder at λ = 240 nm; $\lambda_{max,1}$ = 260 nm; $\lambda_{max,2}$ = 433 nm; Figure S7 left) and **13$_{trans}$** ($\lambda_{max,1}$ = 232 nm; $\lambda_{max,2}$ = 333 nm; $\lambda_{max,3}$ = 443 nm; Figure S7, middle) show typical characteristics for peptides containing azoaromatics of the azobenzene-type, according to the classification of Rau, showing two clearly separated absorption maxima for the low lying n–π* transition and for the π–π* transition [7].

Furthermore, an increasing signal at t_R = 22.8 min (signal e, Figure S5) was detected, which belongs to the diaryl hydrazine **Red-2b**, which is formed upon reduction of the starting material **2b** (ESIMS: *m/z*

= 529.2 ([M + Na]$^+$, calculated: 529.2), Figure S6, right). Due to the disrupted π-delocalization in the hydrazine compound **Red-2b** compared to the corresponding azobenzene **2b**, the UV–vis spectrum lacks the long-wavelength absorption band at λ = 410–550 nm (Figure S7, right). The ligation mixture was stirred overnight and complete consumption of the cysteinyl peptide **3** was detected after 21 h.

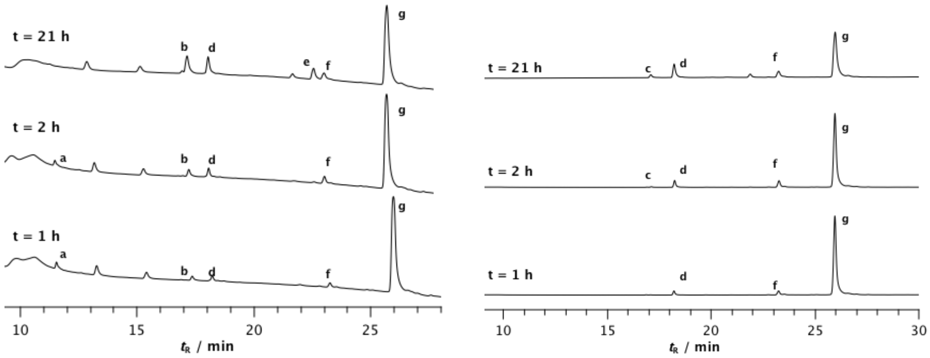

Figure S5: HPLC traces of the reaction course for the synthesis of peptide **13**, detection at 210 nm (left) and 440 nm (right): (a) cysteinyl peptide **3**; (b) thiol leaving group Aatp as disulfide; (c) *cis*-ligation product $\mathbf{13}_{\mathbf{\textit{cis}}}$; (d) *trans*-ligation product $\mathbf{13}_{\mathbf{\textit{trans}}}$; (e) reduced hydrazine form **Red-2b** of the azobenzene thioester **2b**; (f) *cis*-azobenzene thioester $\mathbf{2b}_{\mathbf{\textit{cis}}}$; (g) *trans*-azobenzene thioester $\mathbf{2b}_{\mathbf{\textit{trans}}}$.

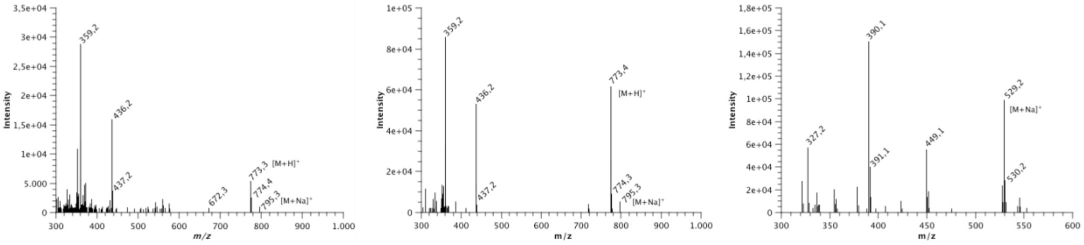

Figure S6: ESIMS spectra of the HPLC signals c ($\mathbf{13}_{\mathbf{\textit{cis}}}$, left), d ($\mathbf{13}_{\mathbf{\textit{trans}}}$, middle), and e (hydrazine form **Red-2b** of thioester **2b**, right).

Reduced hydrazine form **Red-2b** of thioester starting material **2b**:

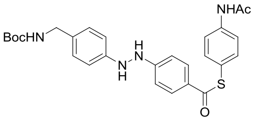

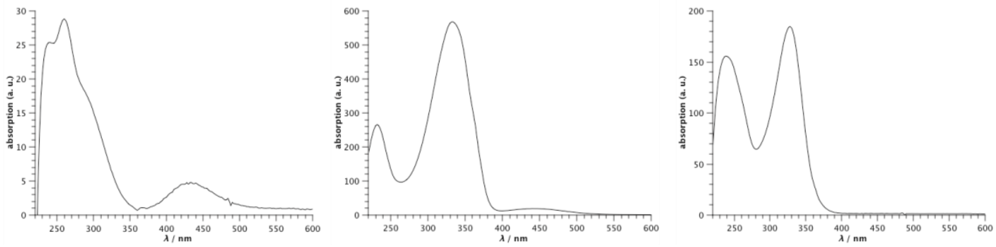

Figure S7: UV–vis spectra: *cis*-ligation product **13***cis* (left), *trans*-ligation product **13***trans* (middle), and of the reduced form (hydrazine) **Red-2b** of starting material **2b** (right).

LC–MS and UV–vis data on the formation of reduced ligation peptide Red-13

The LC–MS analysis of the crude product after extractive workup and lyophilization revealed a mixture of the desired ligation product **13** in its *cis*- and *trans*-form and the reduced diaryl hydrazine peptide **Red-13** (Figure S8). As in the case of the reduced thioester starting material, the lack of a long-wavelength absorption band at λ = 410–550 nm is a sign of the disrupted π-delocalization in the hydrazine form (Figure S8, right). Upon integration of the signals detected at λ = 210 nm, an HPLC-based ratio of the compounds **13***cis/trans*/**Red-13** of 52:48 is determined in the crude product.

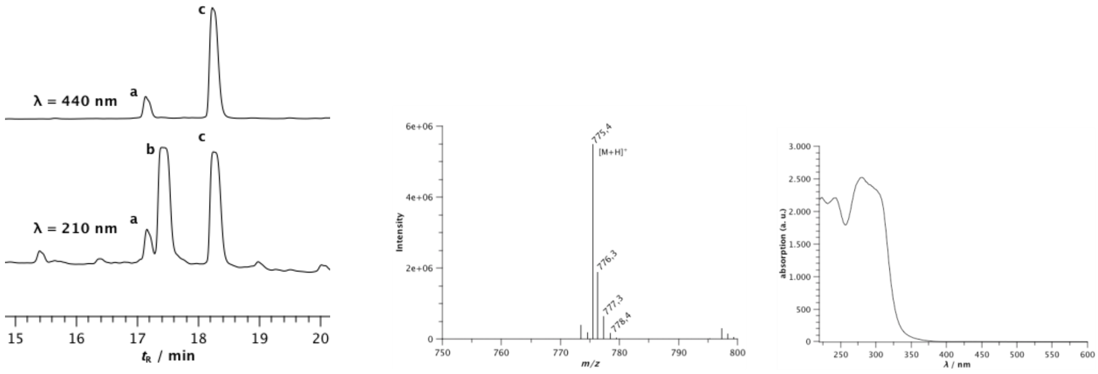

Figure S8: *Left*: HPLC traces of the ligation mixture after extractive workup and lyophilization (HPLC conditions G); (a) *cis*-ligation product **13***cis*; (b) reduced ligation product **Red-13**; (c) *trans*-ligation product **13***trans*. *Middle*: ESIMS spectrum of HPLC signal b. *Right*: UV–vis data of HPLC signal b.

Cysteine-free native chemical ligation

Typical procedure

To a solution of the disulfide of the auxiliary-capped peptide **4** or **5** (1 equiv) in DMF (0.5 mmol/L), TCEP × HCl (5.5 equiv), Na_2HPO_4 (11 equiv) and the photochromic thioester (3.8 equiv, neat) were added, and the pH of the reaction mixture was detected and adjusted with additional amounts of Na_2HPO_4 to pH 7.3 after sonication. The mixture was then stirred at rt under an atmosphere of nitrogen.

After the HPLC analysis of the ligation mixture showed complete conversion of the peptide **4** or **5**, the ligation mixture was diluted with water at 0 °C to obtain a 1:1 mixture of DMF/water and was washed three times with ice-cold diethyl ether and three times with ice-cold ethyl acetate.

Ligation of azobenzene thioester 1b with peptide 4: Peptide 11

11 and **Red-11**:

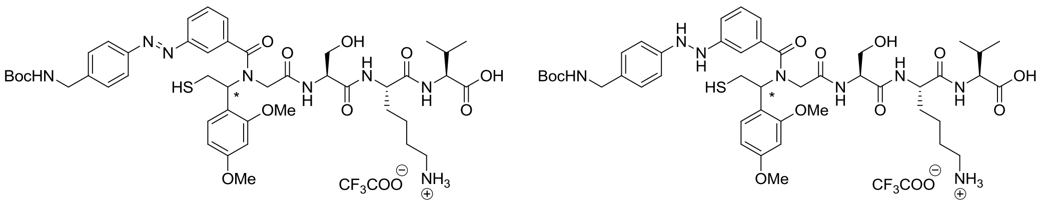

The ligation was carried out with 11 mg of a diastereomeric mixture of the disulfide of peptide **4** (7.9 µmol, 1 equiv) in 16 mL of DMF. TCEP × HCl (12.4 mg, 43.2 µmol, 5.5 equiv), Na_2HPO_4 (12.5 mg, 88.0 µmol, 11 equiv) and the azobenzene thioester **1b** (15.1 mg, 29.9 µmol, 3.8 equiv) were added and the reaction was worked up as described in the typical procedure after complete consumption of peptide **4** (reaction time: 43 h). After lyophilization of the aqueous layer, the crude product was purified by RP-HPLC on an Agilent preparative HPLC using a Zorbax C18 column (21.2 × 150 mm, 5 µm, Agilent) at a flow rate of 20 mL/min with spectrometric monitoring at λ = 210 nm and λ = 440 nm under HPLC conditions D to give the disulfide of the photochromic peptide **11** (7.0 mg, 3.4 µmol, 43%) as a light-yellow solid.

LC–MS and UV–vis data for the ligation course of peptide 11

The HPLC analyses of the ligation time course were performed after filtration of the ligation mixture to remove solid Na_2HPO_4 using HPLC conditions G with spectrometric monitoring at λ = 210 nm (Figure S9, left) and λ = 430 nm (Figure S9, right).

The ligation product eluted as a double peak because of the racemic nature of the auxiliary. Both diastereomers exhibit identical mass and UV–vis spectra. After 23 h, the *cis*-product $\mathbf{11}_{\boldsymbol{cis}}$ (signals c,c'; t_R = 18.3 min, 18.5 min) and the *trans*-product $\mathbf{11}_{\boldsymbol{trans}}$ (signals d,d'; t_R = 19.6, 19.9 min) were detected in considerable amounts (ESIMS: *m/z* ($\mathbf{11}_{\boldsymbol{cis}}$) = 923.6; *m/z* ($\mathbf{11}_{\boldsymbol{trans}}$) = 923.6 ($[M + H]^+$, calculated: 923.4), Figure S10), but at this time a complete conversion of the peptide starting material **4** was not detected. Since small amounts of peptide **4** were also detected after 30 h, stirring of the ligation mixture was continued overnight for a total ligation time of 43 h.

The UV–vis spectra of the ligation products $\mathbf{11}_{\boldsymbol{cis}}$ ($\lambda_{max,1}$ = 236 nm; $\lambda_{max,2}$ = 279 nm; $\lambda_{max,3}$ = 433 nm, Figure S11 left) and $\mathbf{11}_{\boldsymbol{trans}}$ ($\lambda_{max,1}$ = 234 nm; $\lambda_{max,2}$ = 328 nm; $\lambda_{max,3}$ = 439 nm, Figure S11, middle) show typical characteristics for peptides containing azoaromatics of the azobenzene-type, according to the classification of Rau, showing two clearly separated absorption maxima for the low lying n–π* transition and for the π–π* transition [7].

Furthermore, an increasing signal at t_R = 23.5 min (signal f, Figure S9) was detected, which belongs to the diaryl hydrazine **Red-1b**, which is formed upon reduction of the starting material **1b** (ESIMS: *m/z* = 529.3 ($[M + Na]^+$, calculated: 529.2), Figure S10, right). Due to the disrupted π-delocalization in this hydrazine compound **Red-1b** compared to the according azobenzene **1b**, the UV–vis spectrum lacks the long-wavelength absorption band at λ = 410–550 nm (Figure S11, right).

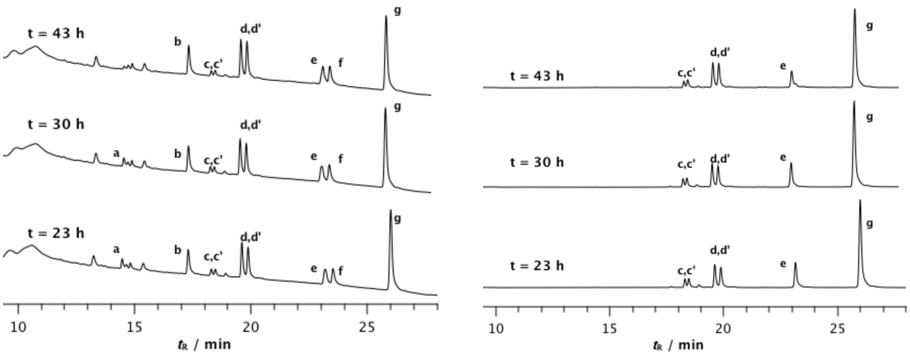

Figure S9: HPLC traces of the reaction course for the synthesis of peptide **11**, detection at 210 nm (left) and 430 nm (right): (a) auxiliary-capped peptide **4**; (b) thiol leaving group Aatp as disulfide; (c,c') diastereomers of the *cis*-ligation product **11**$_{cis}$; (d,d') diastereomers of the *trans*-ligation product **11**$_{trans}$; (e) *cis*-azobenzene thioester **1b**$_{cis}$; (f) reduced hydrazine form **Red-1b** of the azobenzene thioester **1b**; (g) *trans*-azobenzene thioester **1b**$_{trans}$.

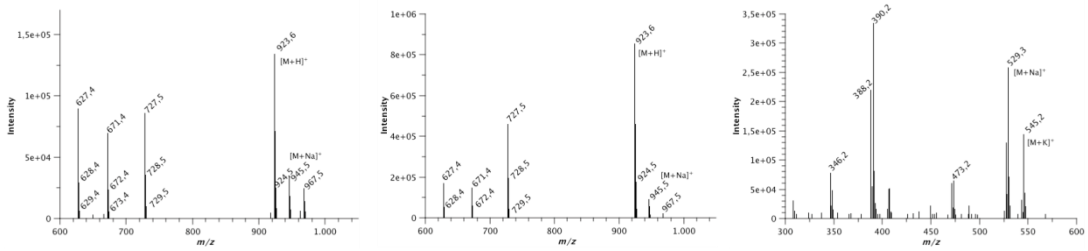

Figure S10: ESIMS spectra of the HPLC signals diastereomers c,c' (**11**$_{cis}$, left), diastereomers d,d' (**11**$_{trans}$, middle) and f (reduced form **Red-1b** of azobenzene thioester **1b**, right).

Reduced hydrazine form **Red-1b** of thioester starting material **1b**:

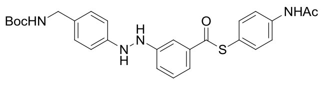

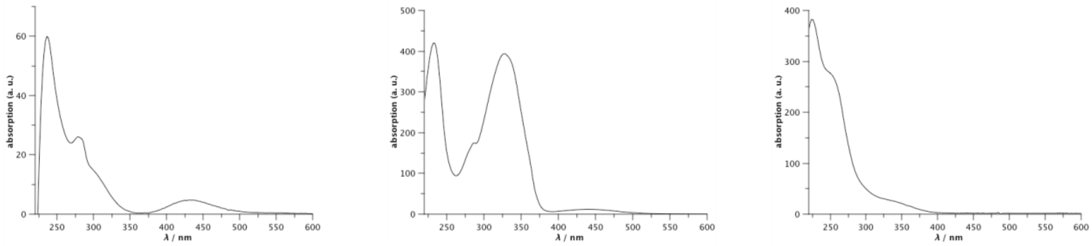

Figure S11: UV–vis spectra: *cis*-ligation product **11**$_{cis}$ (left), *trans*-ligation product **11**$_{trans}$ (middle) and the reduced form **Red-1b** of starting material **1b** (right).

LC–MS and UV–vis data on the formation of reduced peptide Red-11

The LC–MS analysis of the crude product after extractive workup and lyophilization revealed a mixture of the desired ligation product **11** in its *cis*- and *trans*-form and the reduced diaryl hydrazine peptide **Red-11** (Figure S12). As in the case of the reduced thioester starting material, the lack of a long-wavelength absorption band at λ = 410–550 nm is a sign of the disrupted π-delocalization in the hydrazine form (Figure S12, right). Upon integration of the signals detected at λ = 210 nm, an HPLC-based ratio of the compounds $\mathbf{11}_{cis/trans}$/**Red-11** of 97:3 is determined in the crude product.

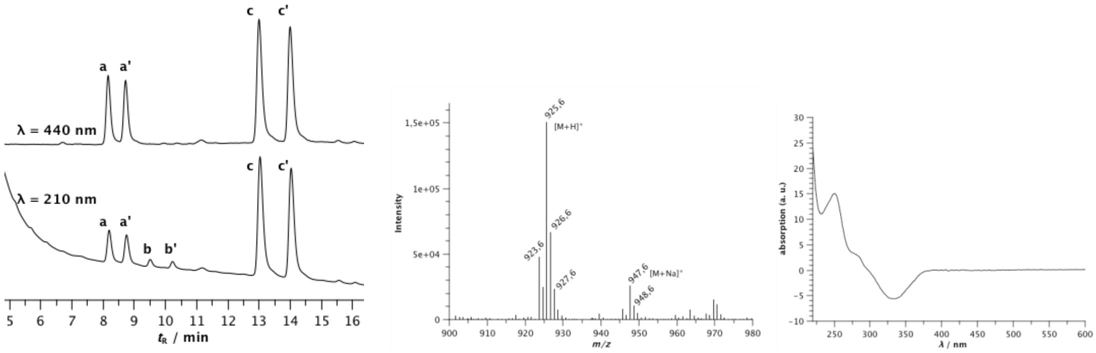

Figure S12: *Left*: HPLC traces of the ligation mixture after extractive workup and lyophilization (HPLC conditions J); (a,a') diastereomers of the *cis*-ligation product $\mathbf{11}_{cis}$; (b,b') diastereomers of the reduced ligation product **Red-11**; (c,c') diastereomers of the *trans*-ligation product $\mathbf{11}_{trans}$. *Middle*: ESIMS spectrum of HPLC signal b. *Right*: UV–vis data of HPLC signal b.

Ligation of azobenzene thioester 1b with peptide 5: Peptide 12

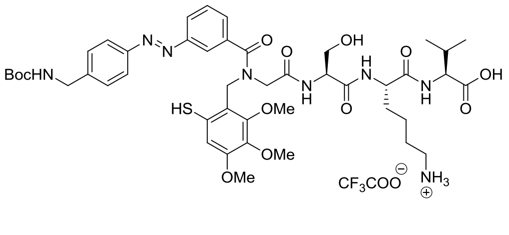

The ligation was carried out with 20 mg of the disulfide of peptide **5** (14.0 µmol, 1 equiv) in 28 mL of DMF. TCEP × HCl (22 mg, 76.8 µmol, 5.5 equiv), Na_2HPO_4 (22 mg, 156.0 µmol, 11 equiv) and the azobenzene thioester **1b** (27 mg, 53.1 µmol, 3.8 equiv) were added, and the reaction was worked up as described in the typical procedure after complete consumption of peptide **5** (reaction time: 23 h). After

lyophilization of the aqueous layer, the crude product was purified by RP-HPLC on an Agilent preparative HPLC using a Zorbax C18 column (21.2 × 150 mm, 5 µm, Agilent) at a flow rate of 20 mL/min with spectrometric monitoring at λ = 210 nm and λ = 440 nm, using HPLC conditions F to give the disulfide of the photochromic peptide **12** (11 mg, 5.3 µmol, 38%) as a light-yellow solid.

LC–MS and UV–vis data for the ligation course of peptide 12

The HPLC analyses of the ligation time course were performed after filtration of the ligation mixture to remove solid Na_2HPO_4 using HPLC conditions G with spectrometric monitoring at λ = 210 nm (Figure S13, left) and λ = 430 nm (Figure S13, right). The formation of the *cis*-product $\mathbf{12}_{cis}$ (signal c, t_R = 18.0 min) and the *trans*-product $\mathbf{12}_{trans}$ (signal d, t_R = 19.5 min) was already detected after 1 h (ESIMS: *m/z* ($\mathbf{12}_{cis}$) = 939.5; *m/z* ($\mathbf{12}_{trans}$) = 939.5 ($[M + H]^+$, calculated: 939.4), Figure S14). The *cis*-configured ligation product $\mathbf{12}_{cis}$ was only detected in very small amounts. Therefore, the analysis of the UV–vis spectrum is difficult (Figure S15, left), but the according spectrum of the *trans*-configured ligation product $\mathbf{12}_{trans}$ ($\lambda_{max,1}$ ≈ 220 nm; $\lambda_{max,2}$ = 328 nm; $\lambda_{max,3}$ = 440 nm, Figure S15, right) shows typical characteristics for peptides containing azoaromatics of the azobenzene-type, according to the classification of Rau, showing two clearly separated absorption maxima for the low lying n–π* transition and for the π–π* transition [7].

The ligation mixture was stirred overnight and complete consumption of the auxiliary-capped peptide **5** was detected after a total of 23 h.

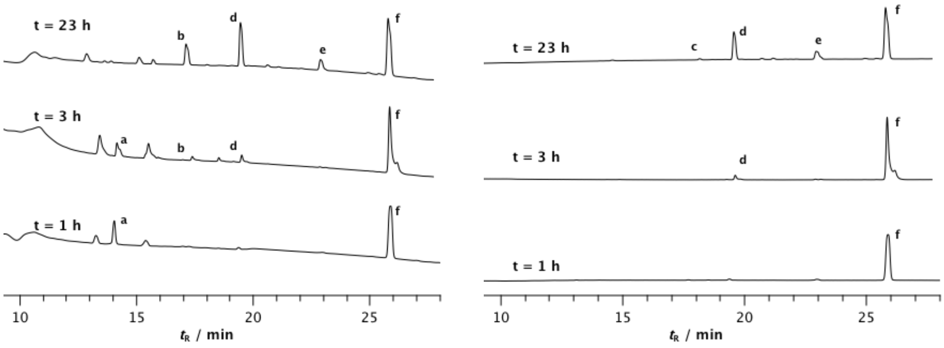

Figure S13: HPLC traces of the reaction course for the synthesis of peptide **12**, detection at 210 nm (left) and 430 nm (right): (a) auxiliary-capped peptide **5**; (b) thiol leaving group Aatp as disulfide; (c) *cis*-ligation product $\mathbf{12}_{cis}$; (d) *trans*-ligation product $\mathbf{12}_{trans}$; (e) *cis*-azobenzene thioester $\mathbf{1b}_{cis}$; (f) *trans*-azobenzene thioester $\mathbf{1b}_{trans}$.

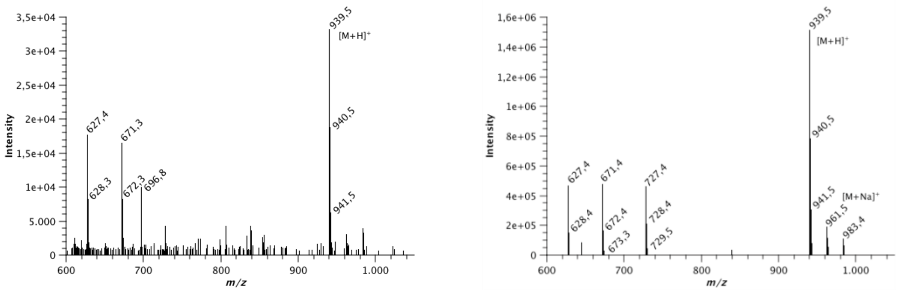

Figure S14: ESIMS spectra: HPLC signals c (**12**$_{cis}$, left) and d (**12**$_{trans}$, right).

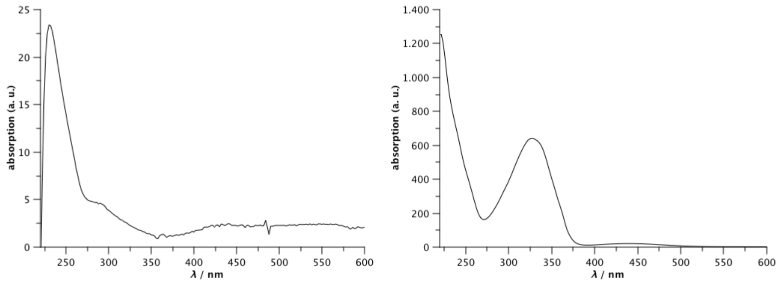

Figure S15: UV–vis spectra: *cis*-ligation product **12**$_{cis}$ (left) and *trans*-ligation product **12**$_{trans}$ (right).

The LC–MS analysis of the crude product after extractive workup and lyophilization revealed a mixture of the desired ligation product **12** in its *cis*- and *trans*-form, but no reduced diaryl hydrazine peptide **Red-12** (Figure S16).

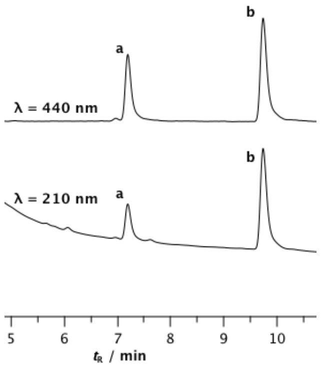

Figure S16: HPLC traces of the ligation mixture after extractive workup and lyophilization (HPLC conditions J): (a) *cis*-ligation product **12**$_{cis}$; (b) *trans*-ligation product **12**$_{trans}$.

Ligation of azobenzene thioester 2b with peptide 4: Peptide 14

14 and **Red-14**:

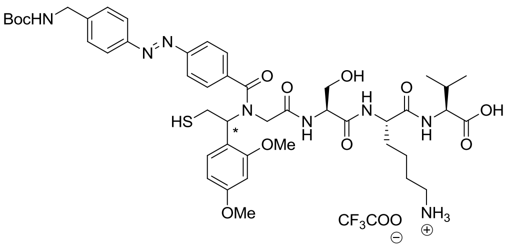

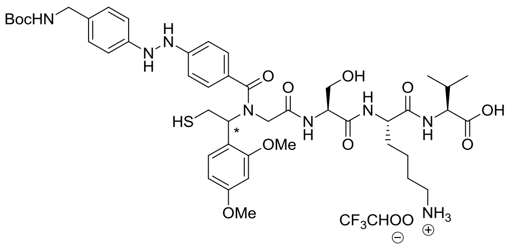

The ligation was carried out with 8.0 mg of a diastereomeric mixture of the disulfide of peptide **4** (5.7 µmol, 1 equiv) in 11 mL of DMF. TCEP × HCl (9.0 mg, 31.4 µmol, 5.5 equiv), Na_2HPO_4 (9.1 mg, 64.0 µmol, 11 equiv) and the azobenzene thioester **2b** (11.0 mg, 21.7 µmol, 3.8 equiv) were added, and the reaction was worked up as described in the typical procedure after complete consumption of peptide **4** (reaction time: 43 h). LC–MS analysis after lyophilization showed, that the obtained light-yellow solid (4.1 mg) consisted of **14** and **Red-14**; ratio **14**/**Red-14** 18:82 (21:79 upon reinvestigation). The crude product was purified by RP-HPLC on an Agilent preparative HPLC using a Zorbax C18 column (21.2 × 150 mm, 5 µm, Agilent) at a flow rate of 20 mL/min with spectrometric monitoring at λ = 210 nm and λ = 440 nm under HPLC conditions D to give the disulfide of the photochromic peptide **14** (2.0 mg, 1.0 µmol, 17%) as a light-yellow solid and the disulfide of the diaryl hydrazine peptide **Red-14** (3.1 mg, 1.5 µmol, 26%) as a colorless solid, which was obtained in a colorless solution and turned upon exposure to air into the desired product **14**.

LC–MS and UV–vis data for the ligation course of peptide 14

The HPLC analyses of the ligation time course were performed after filtration of the ligation mixture to remove solid Na_2HPO_4 using HPLC conditions H with spectrometric monitoring at λ = 210 nm (Figure S17, left) and λ = 430 nm (Figure S17, right). The ligation product eluted as a double peak because of the racemic nature of the auxiliary. Both diastereomers exhibit identical mass and UV–vis spectra. After 27 h, the *cis*-product $\mathbf{14}_{\mathbf{\mathit{cis}}}$ (signals c,c’; t_R = 21.9 min, 22.4 min) and the *trans*-product

14_{trans} (signal d,d'; t_R = 24.3, 24.9 min) were detected in considerable amounts (ESIMS: *m/z* (**14_{cis}**) = 923.6; *m/z* (**14_{trans}**) = 923.5 ($[M + H]^+$, calculated: 923.4), Figure S18), but complete conversion of the peptide starting material **4** was not detected at this time. Therefore, stirring of the ligation mixture was continued overnight for a total ligation time of 43 h.

The UV–vis spectra of the ligation products **14_{cis}** ($\lambda_{max,1}$ = 229 nm; $\lambda_{max,2}$ = 280 nm; $\lambda_{max,3}$ = 432 nm, Figure S19 left) and **14_{trans}** ($\lambda_{max,1}$ = 231 nm; $\lambda_{max,2}$ = 332 nm; $\lambda_{max,3}$ = 440 nm, Figure S19, middle) show typical characteristics for peptides containing azoaromatics of the azobenzene-type, according to the classification of Rau, showing two clearly separated absorption maxima for the low lying n–π* transition and for the π–π* transition [7].

Furthermore, an increasing signal at t_R = 29.3 min (signal e, Figure S17) was detected, belonging to the diaryl hydrazine **Red-2b**, which is formed upon reduction of the starting material **2b** (ESIMS: *m/z* = 529.2 ($[M + Na]^+$, calculated: 529.2), Figure S18, right). Due to the disrupted π-delocalization in this hydrazine compound **Red-2b** compared to the corresponding azobenzene **2b**, the UV–vis spectrum lacks the long-wavelength absorption band at λ = 410–550 nm (Figure S19, right).

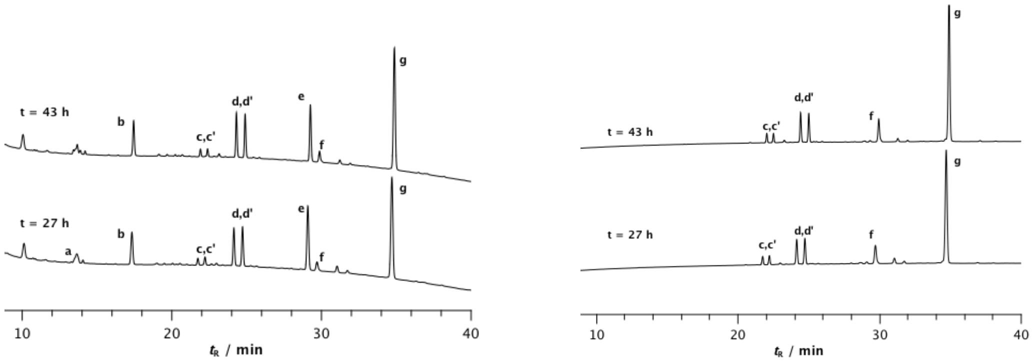

Figure S17: HPLC traces of the reaction course for the synthesis of peptide **14**, detection at 210 nm (left) and 430 nm (right): (a) auxiliary-capped peptide **4**; (b) thiol leaving group Aatp as disulfide; (c,c') diastereomers of the *cis*-ligation product **14_{cis}**; (d,d') diastereomers of the *trans*-ligation product **14_{trans}**; (e) reduced hydrazine form **Red-2b** of the azobenzene thioester **2b**; (f) *cis*-azobenzene thioester **$2b_{cis}$**; (g) *trans*-azobenzene thioester **$2b_{trans}$**.

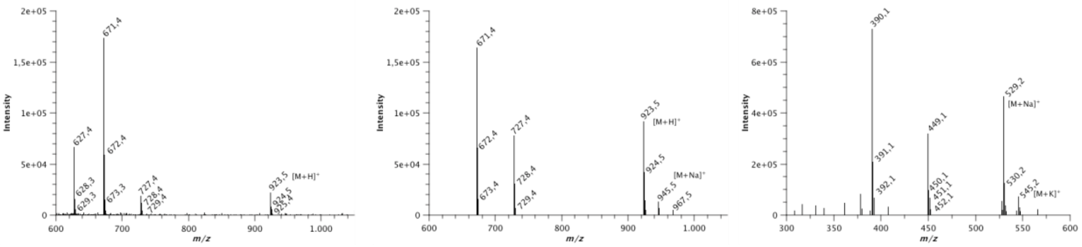

Figure S18: ESIMS spectra of the HPLC signals of the diastereomers c,c' ($\mathbf{14}_{cis}$, left), and d,d' ($\mathbf{14}_{\mathrm{trans}}$, middle), and of the hydrazine form **Red-2b** of the azobenzene thioester **2b** (right).

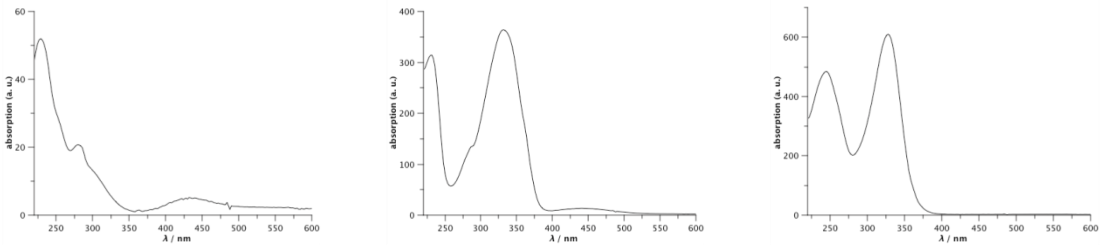

Figure S19: UV–vis spectra: *cis*-ligation product $\mathbf{14}_{cis}$ (left), *trans*-ligation product $\mathbf{14}_{trans}$ (middle), and the reduced form **Red-2b** of the starting material **2b** (right).

LC–MS and UV–vis data on the formation of reduced peptide Red-14

The LC–MS analysis of the crude product after extractive workup and lyophilization revealed a mixture of the desired ligation product **14** and the reduced diaryl hydrazine peptide **Red-14** (Figure S20). As in the case of the reduced thioester **Red-2b**, the lack of a long-wavelength absorption band at λ = 410–550 nm is a sign of the disrupted π-delocalization in the hydrazine **Red-2b** (Figure S20, right). Upon integration of the signals detected at λ = 210 nm, an HPLC-based ratio of the compounds **14**/**Red-14** of 18:82 is determined in the crude product.

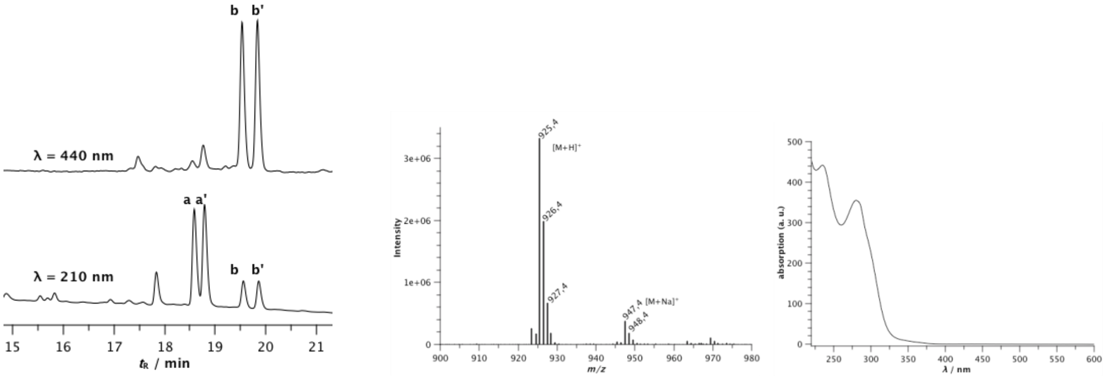

Figure S20: *Left*: HPLC traces of the ligation mixture after extractive workup and lyophilization (HPLC conditions G): (a,a') diastereomers of the reduced ligation product **Red-14**; (b,b') diastereomers of the *trans*-ligation product $\mathbf{14}_{trans}$. *Middle*: ESIMS spectrum of HPLC signal a. *Right*: UV–vis spectrum of HPLC signal a.

Ligation of azobenzene thioester 2b with peptide 5: Peptide 15

15 and **Red-15**:

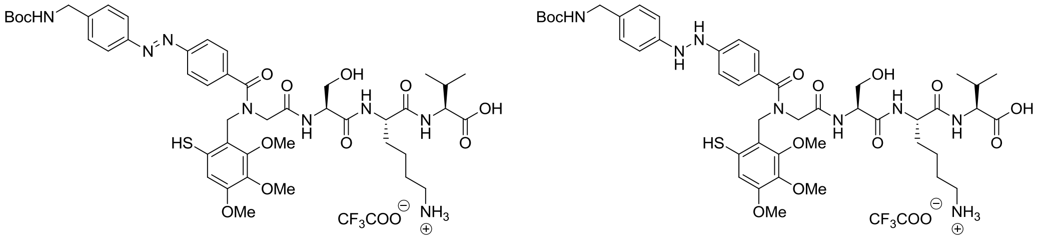

The ligation was carried out with 15.0 mg of the disulfide of peptide **5** (10.5 µmol, 1 equiv) in 21 mL of DMF. TCEP × HCl (16.5 mg, 57.6 µmol, 5.5 equiv), Na_2HPO_4 (16.7 mg, 118.0 µmol, 11 equiv) and the azobenzene thioester **2b** (20 mg, 39.9 µmol, 3.8 equiv) were added, and the reaction was worked up as described in the typical procedure after complete consumption of peptide **5** (reaction time: 24 h). After lyophilization of the aqueous layer, the crude product was purified by RP-HPLC on an Agilent preparative HPLC using a Zorbax C18 column (21.2 × 150 mm, 5 µm, Agilent) at a flow rate of 20 mL/min with spectrometric monitoring at λ = 210 nm and λ = 440 nm, under HPLC conditions D to give the disulfide of the photochromic peptide **15** (10.0 mg, 4.7 µmol, 45%) as a light-yellow solid.

LC–MS and UV–vis data for the ligation course of peptide 15

The HPLC analyses of the ligation time course were performed after filtration of the ligation mixture to remove solid Na_2HPO_4 under HPLC conditions G with spectrometric monitoring at λ = 210 nm (Figure S21, left) and λ = 430 nm (Figure S21, right).

The formation of the *cis*-product $\mathbf{15}_{cis}$ (signal c, t_R = 18.2 min) and the *trans*-product $\mathbf{15}_{trans}$ (signal d, t_R = 19.3 min) was detected in considerable amounts after five hours (ESIMS: *m/z* ($\mathbf{15}_{cis}$) = 939.5; *m/z* ($\mathbf{15}_{trans}$) = 939.5 ($[M + H]^+$, calculated: 939.4), Figure S22).

The UV–vis spectra of the ligation products $\mathbf{15}_{cis}$ ($\lambda_{max,1}$ = 231 nm; $\lambda_{max,2}$ = 436 nm; shoulders at λ = 245 nm and 281 nm, Figure S23 left) and $\mathbf{15}_{trans}$ ($\lambda_{max,1}$ = 222 nm; $\lambda_{max,2}$ = 331 nm; $\lambda_{max,3}$ = 439 nm, Figure S23, right) show typical characteristics for peptides containing azoaromatics of the azobenzene-type, according to the classification of Rau, showing two clearly separated absorption maxima for the low lying n–π* transition and for the π–π* transition [7].

As observed in the other ligation reactions with azobenzene thioester **2b**, the formation of a diaryl hydrazine **Red-2b** (signal e, t_R = 22.5 min) was detected also in this case.

The ligation mixture was stirred overnight and complete consumption of the auxiliary-capped peptide **5** was detected after a total reaction time of 24 h.

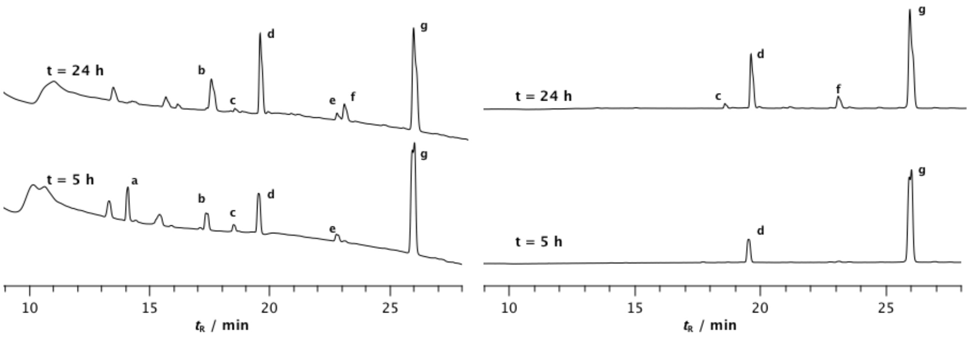

Figure S21: HPLC traces of the reaction course for the synthesis of peptide **15**, detection at 210 nm (left) and 430 nm (right): (a) auxiliary-capped peptide **5**; (b) thiol leaving group Aatp as disulfide; (c) *cis*-ligation product $\mathbf{15}_{cis}$; (d) *trans*-ligation product $\mathbf{15}_{trans}$; (e) reduced hydrazine form **Red-2b** of the azobenzene thioester **2b**; (f) *cis*-azobenzene thioester $\mathbf{2b}_{cis}$; (g) *trans*-azobenzene thioester $\mathbf{2b}_{trans}$.

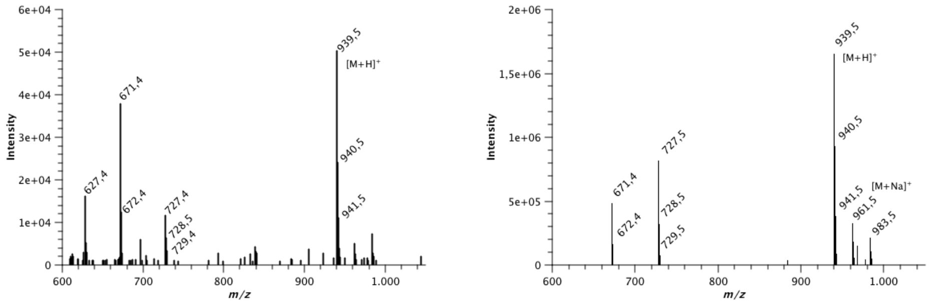

Figure S22: ESIMS spectra: HPLC signals c (**15***cis*, left) and d (**15trans**, right).

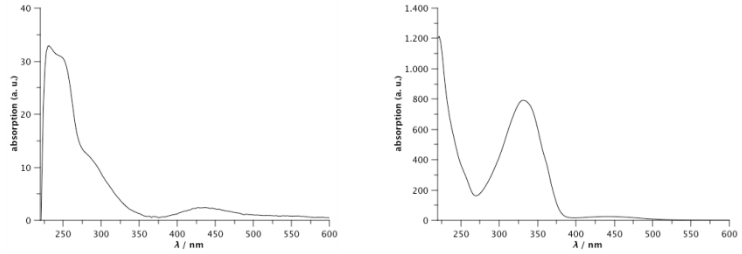

Figure S23: UV–vis spectra: *cis*-ligation product **15***cis* (left) and *trans*-ligation product **15***trans* (right).

LC–MS and UV–vis data on the formation of reduced peptide Red-15

The LC–MS analysis of the crude product after extractive workup and lyophilization revealed a mixture of the desired ligation product **15** and the reduced diaryl hydrazine peptide **Red-15** (Figure S24). The lack of a long-wavelength absorption band at λ = 410–550 nm is a sign of the disrupted π-delocalization in the hydrazine **Red-15** (Figure S24, right). Upon integration of the signals detected at λ = 210 nm, a HPLC-based ratio of the compounds **15**/**Red-15** of 90:10 is determined in the crude product.

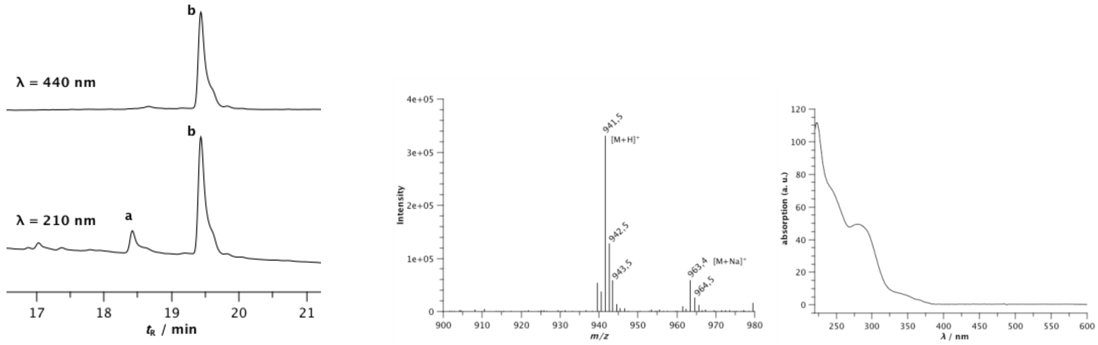

Figure S24: *Left*: HPLC traces of the ligation mixture after extractive workup and lyophilization (HPLC conditions G); (a) reduced ligation product **Red-15**; (b) *trans*-ligation product **15***trans*. *Middle*: ESIMS spectrum of HPLC signal a. *Right*: UV–vis spectrum of HPLC signal a.

TFA-mediated deprotection and cleavage of the auxiliaries

General procedure

A solution of the photochromic peptide **11**, **12** or **14** in 90% aq. TFA (1 mmol/L) was shaken gently for 3.5 h at rt under an atmosphere of nitrogen. The cleavage mixture was concentrated in vacuo by azeotropic distillation with hexane (three times) and the residue was dissolved in water (0.2 mmol/L) and washed three times with ice-cold ethyl acetate. After lyophilization of the aqueous layer, the crude product was purified by reversed-phase high-performance liquid chromatography (RP-HPLC) on an Agilent preparative HPLC using a Zorbax C18 column (21.2 × 150 mm, 5 µm, Agilent) at a flow rate of 20 mL/min with spectrometric monitoring at λ = 210 nm and λ = 440 nm.

Synthesis of peptide 16

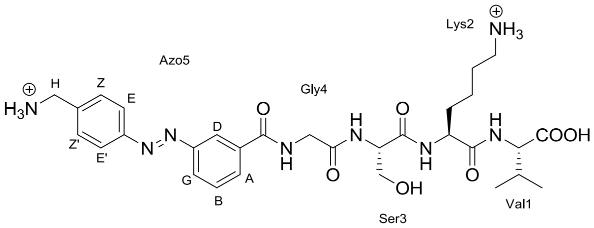

By cleavage of the auxiliary from peptide 11

The cleavage was carried out with 13 mg (12.5 µmol) of peptide **11** as described in the general procedure to give 10 mg (11.7 µmol, 94%) of peptide **16** after purification by RP-HPLC (HPLC conditions C) as a light-yellow solid.

By cleavage of the auxiliary from peptide 12

The cleavage was carried out with 7 mg (6.6 µmol) of peptide **12** as described in the general procedure to give 5 mg (5.9 µmol, 89%) of peptide **16** after purification by RP-HPLC (HPLC conditions C) as a light-yellow solid.

^{1}H NMR (d_6-DMSO, 500 MHz): δ = 9.06 (s, 1H, Gly4-NH), 8.39 (s, 1 H, Azo5-C$H_{(D)}$), 8.18 (d, ^3J = 7.1 Hz, 1H, Lys2-NH), 8.07–8.03 (m, 3H, Azo5-C$H_{(A)}$, Azo5-C$H_{(G)}$, Ser3-NH), 7.97 (d, ^3J = 8.0 Hz, 2H, Azo5-C$H_{(E,E')}$), 7.75–7.66 (m, 4H, Azo5-C$H_{(B)}$, Azo5-C$H_{(Z,Z')}$, Val1-NH), 4.39–4.37 (m, 1H, Ser3-

C$H_{(\alpha)}$), 4.26–4.25 (m, 1H, Lys2-C$H_{(\alpha)}$), 4.14 (s, 2H, Azo5-C$H_{2(H)}$), 4.01–3.93 (m, 3H, Gly4-C$H_{(\alpha 1,2)}$, Val1-C$H_{(\alpha)}$), 3.64–3.56 (m, 2H, Ser3-C$H_{2(\beta)}$), 2.76–2.73 (m, 2H, Lys2-C$H_{2(\varepsilon)}$), 2.02–1.98 (m, 1H, Val1-C$H_{(\beta)}$), 1.74–1.53 (m, 4H, Lys2-C$H_{(\beta 1,2)}$, Lys2-C$H_{(\delta 1,2)}$)), 1.36–1.35 (m, 2H, Lys2-C$H_{2(\gamma)}$), 0.82 (d, ^3J = 6.6 Hz, 6H, Val1-C$H_{3(\gamma)}$) ppm.

Synthesis of peptide 17

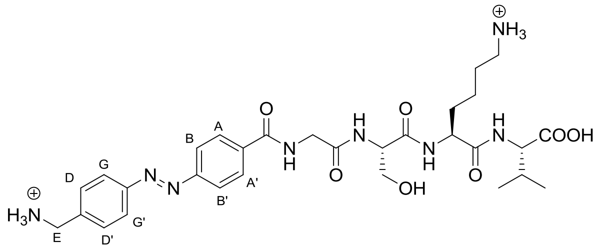

The cleavage was carried out with 7.5 mg (7.2 µmol) of peptide **14** as described in the general procedure to give 5 mg (5.8 µmol, 81%) of peptide **17** after purification by RP-HPLC (HPLC-conditions E) as a light-yellow solid.

^{1}H NMR (d_6-DMSO, 400 MHz): δ = 9.03–8.99 (m, 1H, Gly4-NH), 8.16–8.09 (m, 4 H), 8.00–7.98 (m, 3H), 7.84–7.81 (m, 1H), 7.70 (d, ^3J = 7.8 Hz, 2H, Azo5-C$H_{(D,D')}$), 5.09 (br. s, 1H, Ser3-OH), 4.38–4.32 (m, 1H, Ser3-C$H_{(\alpha)}$), 4.17 (s, 2H, Azo5-C$H_{2(E)}$), 4.17–4.04 (m, 3H, Lys2-C$H_{(\alpha))}$, Gly4-C$H_{(\alpha 1,2)}$, Val1-C$H_{(\alpha)}$), 3.62–3.53 (m, 2H, Ser3-C$H_{2(\beta)}$), 2.77–2.73 (m, 2H, Lys2-C$H_{2(\varepsilon)}$), 2.01–1.99 (m, 1H, Val1-C$H_{(\beta)}$), 1.78–1.23 (m, 11H, Lys2-C$H_{(\beta 1,2)}$, Lys2-C$H_{(\delta 1,2)}$, Lys2-C$H_{2(\gamma)}$), 0.84 (d, ^3J = 6.6 Hz, 6H, Val1-C$H_{3(\gamma)}$) ppm.

Analytical data of synthesized peptides

Table 1: Analytical data of synthesized peptides[a].

**Compound**	**R$_{t,cis}$**[b] **[min]**	**R$_{t,trans}$**[b] **[min]**	**HRMS–ESI**[c]
Peptide **3** (disulfide)	–	11.5	*m/z* [M + 2H]$^{2+}$ calcd for $[C_{34}H_{66}N_{10}O_{12}S_2]^{2+}$: 435.2146 found: 435.2142
Peptide **4** (disulfide)	–	14.0	*m/z* [M + 2H]$^{2+}$ calcd for $[C_{52}H_{86}N_{10}O_{16}S_2]^{2+}$: 585.2827 found: 585.2824
Peptide **5** (disulfide)	–	14.5	*m/z* [M + 2H]$^{2+}$ calcd for $[C_{52}H_{86}N_{10}O_{18}S_2]^{2+}$: 601.2776 found: 601.2777
Peptide **10** (disulfide)	18.3	18.8	*m/z* [M + H]$^{2+}$ calcd for $[C_{72}H_{104}N_{16}O_{18}S_2]^{2+}$: 772.3572 found: 772.3560
Peptide **13** (disulfide)	17.1	18.2	*m/z* [M + H]$^{2+}$ calcd for $[C_{72}H_{104}N_{16}O_{18}S_2]^{2+}$: 772.3572 found: 772.3560
Peptide **14**	18.4[d] 18.6[d]	19.6[d] 19.9[d]	*m/z* [M + H]$^{+}$ calcd for $[C_{45}H_{63}N_8O_{11}S]^{+}$: 923.4332 found: 923.4319
Peptide **Red-14**	–	27.1[d,e] 27.5[d,e]	*m/z* [M + H]$^{+}$ calcd for $[C_{45}H_{65}N_8O_{11}S]^{+}$: 925.4488 found: 925.4472
Peptide **11**	18.3[d] 18.5[d]	19.6[d] 19.9[d]	*m/z* [M + H]$^{+}$ calcd for $[C_{45}H_{63}N_8O_{11}S]^{+}$: 923.4332 found: 923.4326
Peptide **12** (disulfide)	18.0	19.5	*m/z* [M + 2H]$^{2+}$ calcd for $[C_{90}H_{124}N_{16}O_{24}S_2]^{2+}$: 938.4202 found: 938.4201
Peptide **15** (disulfide)	18.2	19.3	*m/z* [M + 2H]$^{2+}$ calcd for $[C_{90}H_{124}N_{16}O_{24}S_2]^{2+}$: 938.4202 found: 938.4200
Peptide **16**	13.1	13.6	*m/z* [M + H]$^{+}$ calcd for $[C_{30}H_{43}N_8O_7]^{+}$: 627.3249 found: 627.3244
Peptide **17**	12.5	12.8	*m/z* [M + H]$^{+}$ calcd for $[C_{30}H_{43}N_8O_7]^{+}$: 627.3249 found: 627.3244

[a]Reduced peptides **Red-10, Red-13, Red-11** and **Red-15** were detected during HPLC monitoring after extractive workup and lyophilization, but these peptides were not isolated and analyzed by HRMS. [b]Analytical HPLC, conditions G. [c]Symmetric disulfides of peptides **3, 4**, **5**, and **10**–**15** were obtained after HPLC purification. If a mixture of the appropriate free thiol and the disulfide was observed during HPLC/HRMS measurements (peptides **11**, **14** and **Red-14**), then only the analytical data of the free thiol are shown. [d]Retention time of one of both diastereomers. [e]Analytical HPLC, conditions H.

NMR spectra of compounds 1a, 2a, 1b, 2b, 3–5 and 10, 13, 16 and 17

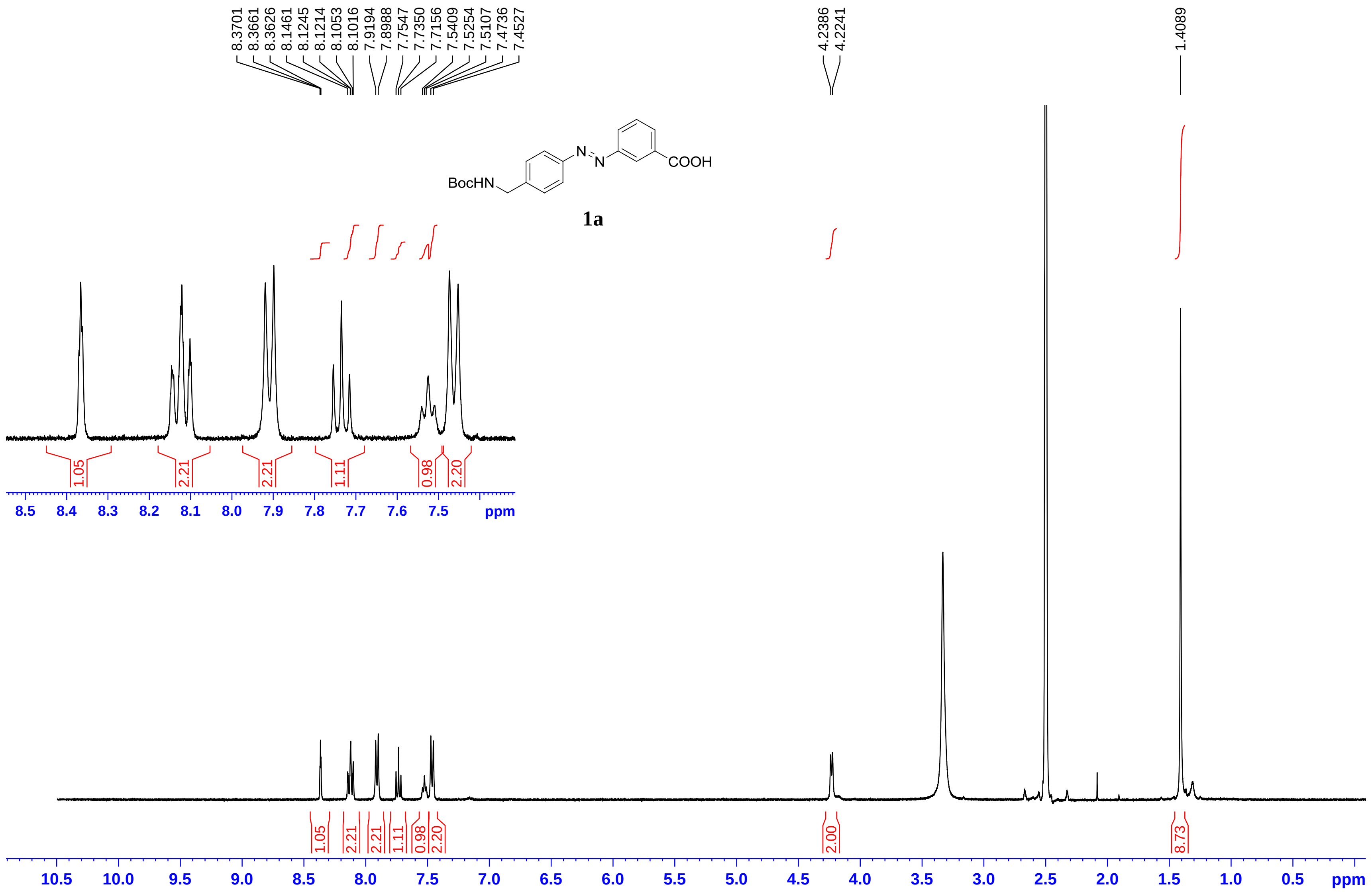

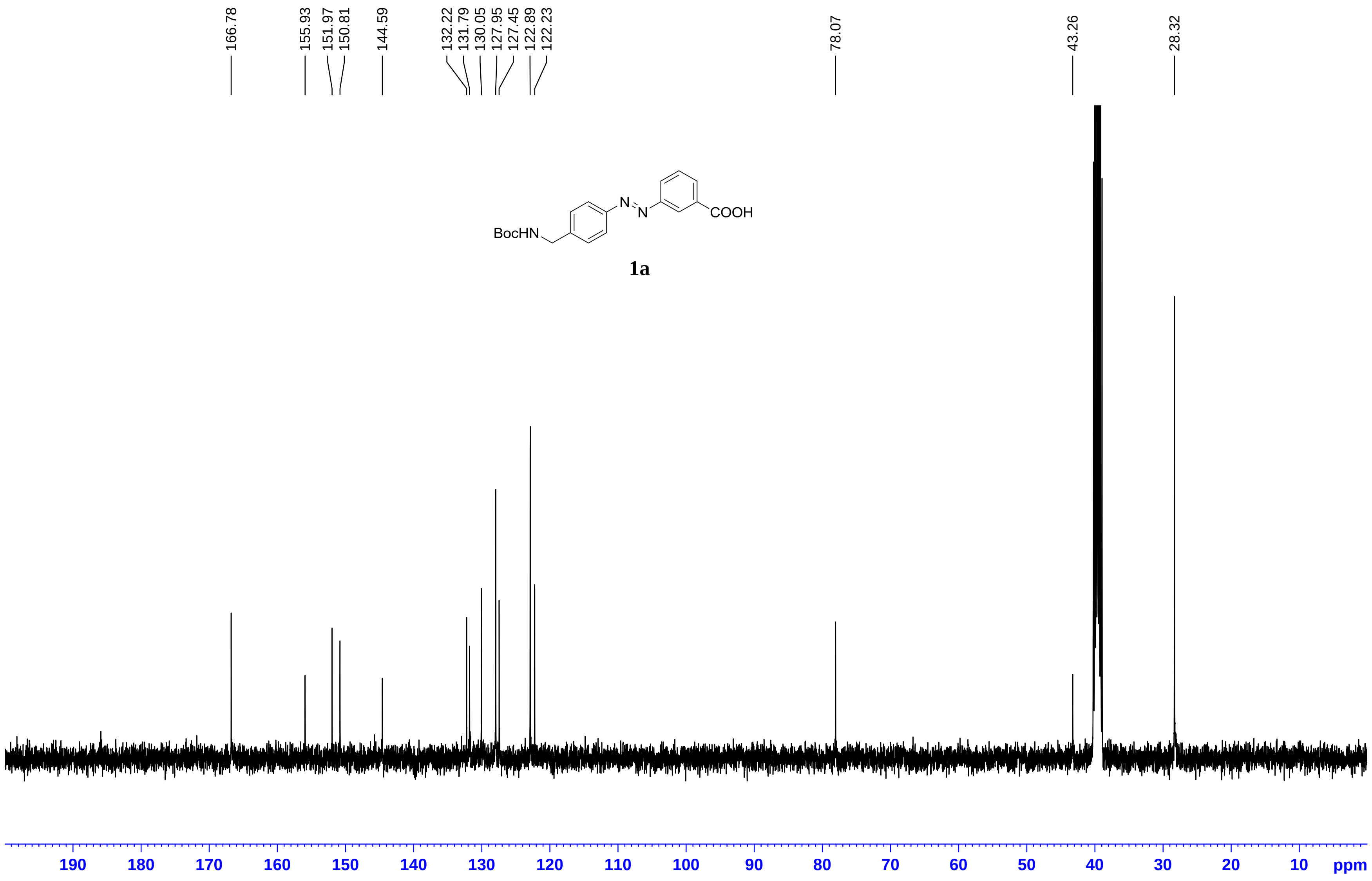
166.78
155.93
151.97
150.81
144.59
132.22
131.79
130.05
127.95
127.45
122.89
122.23
78.07
43.26
28.32
BocHN
N
N
COOH
1a
190
180
170
160
150
140
130
120
110
100
90
80
70
60
50
40
30
20
10
ppm

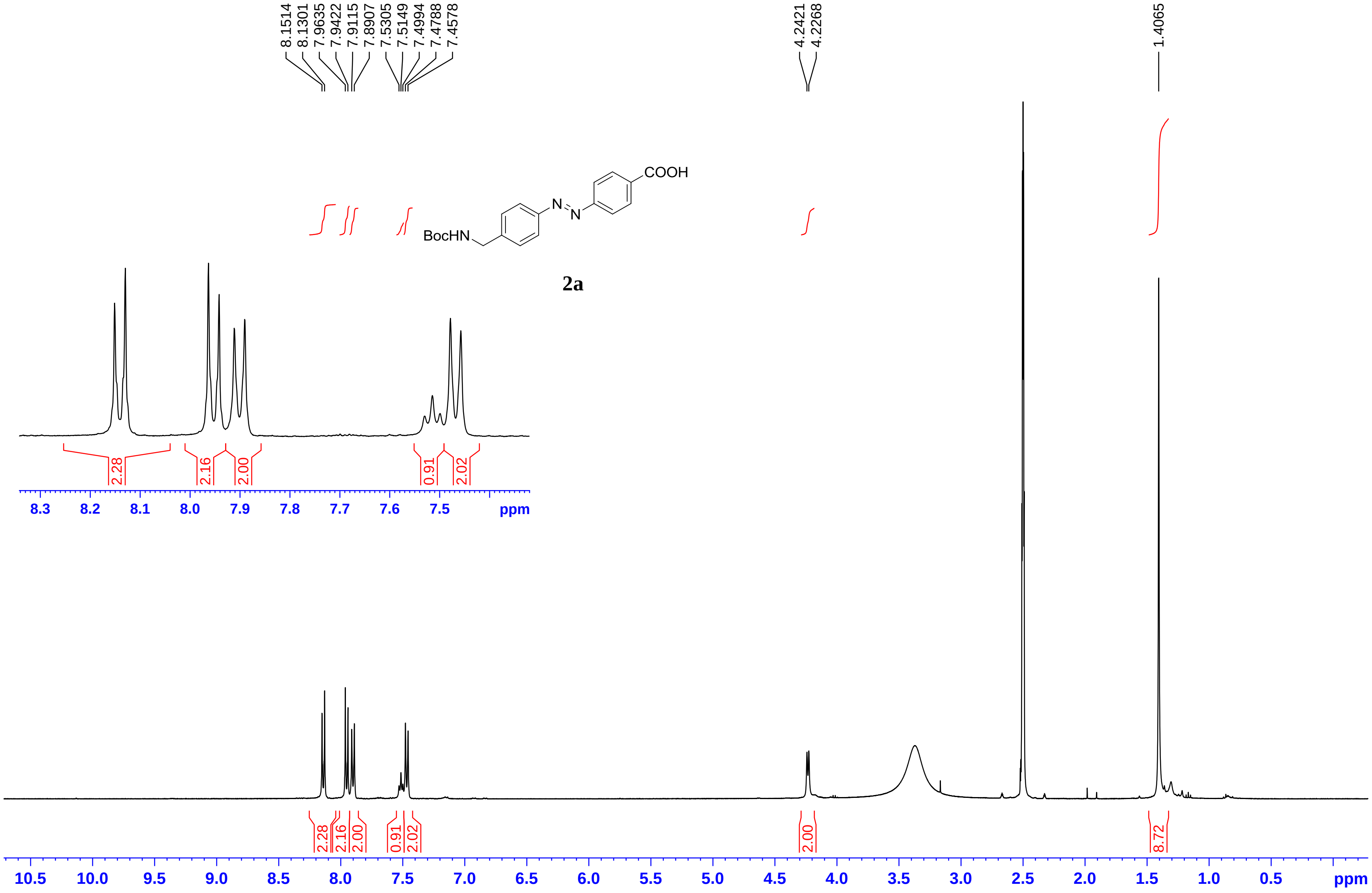
8.1514
8.1301
7.9635
7.9422
7.9115
7.8907
7.5305
7.5149
7.4994
7.4788
7.4578
4.2421
4.2268
1.4065
COOH
N
N
BocHN
2a
2.28
2.16
2.00
0.91
2.02
8.72
8.3 8.2 8.1 8.0 7.9 7.8 7.7 7.6 7.5 ppm
10.5 10.0 9.5 9.0 8.5 8.0 7.5 7.0 6.5 6.0 5.5 5.0 4.5 4.0 3.5 3.0 2.5 2.0 1.5 1.0 0.5 ppm

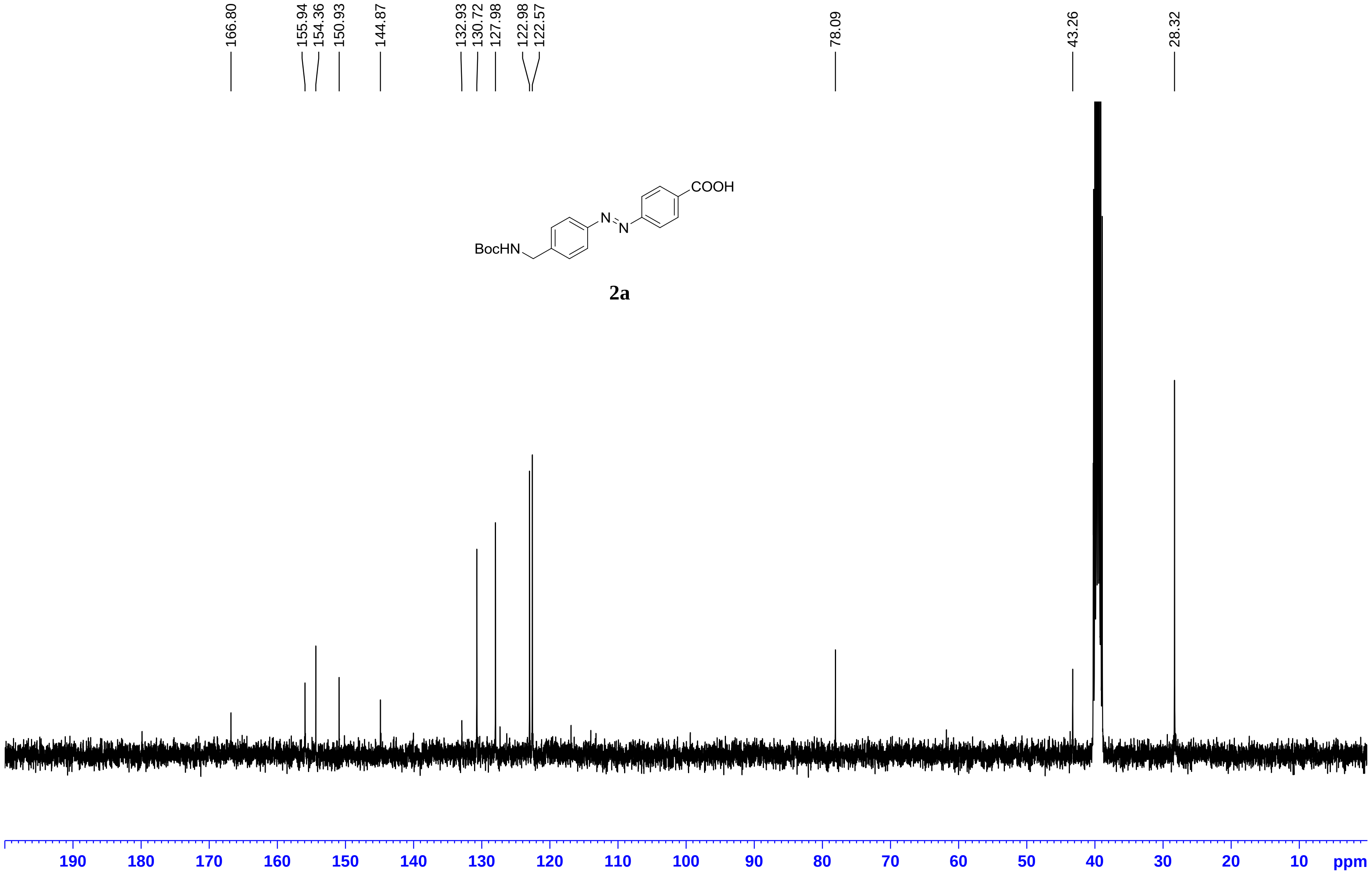
166.80
155.94
154.36
150.93
144.87
132.93
130.72
127.98
122.98
122.57
78.09
43.26
28.32
COOH
N
N
BocHN
2a
190
180
170
160
150
140
130
120
110
100
90
80
70
60
50
40
30
20
10
ppm

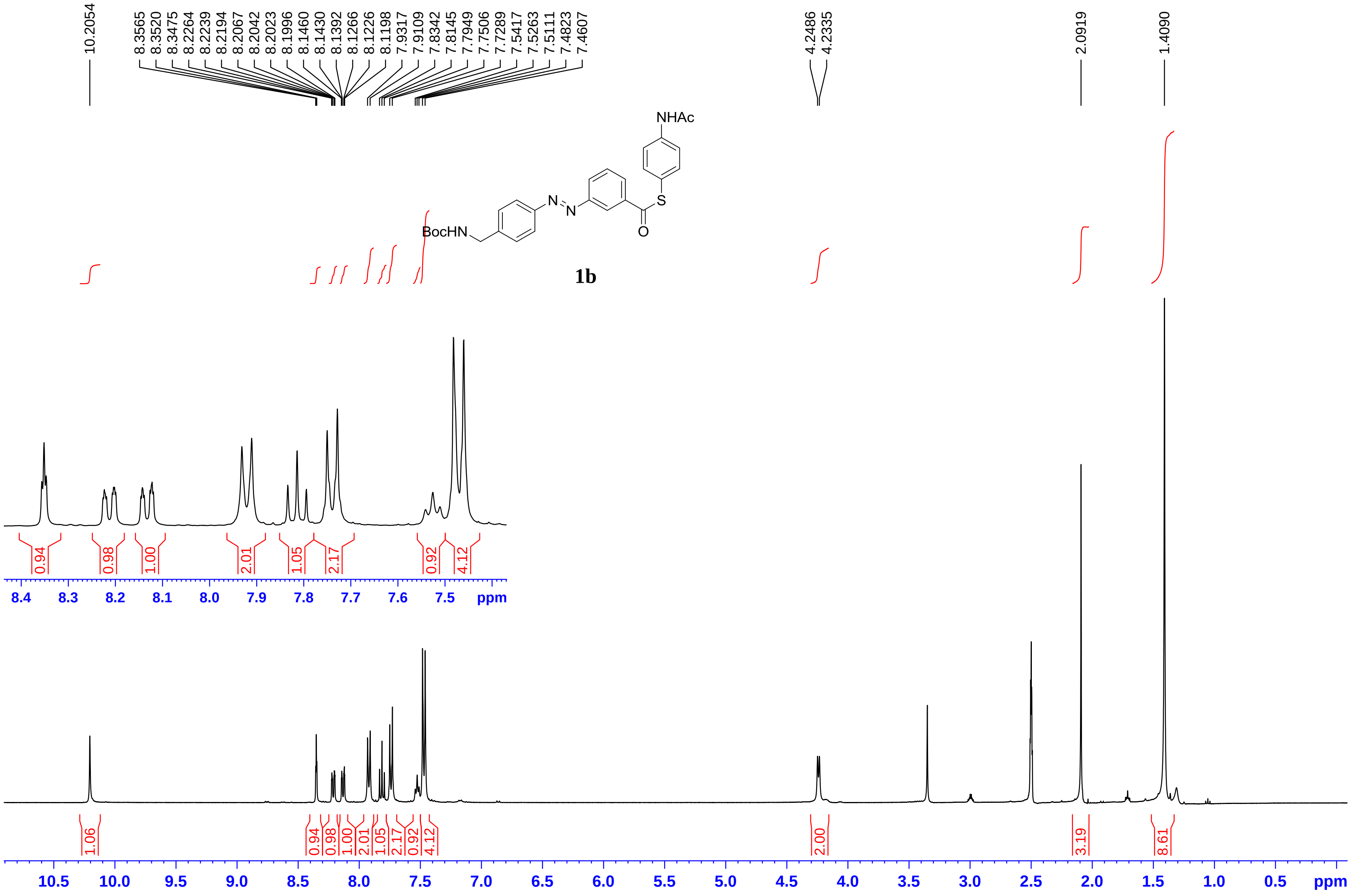
10.2054
8.3565
8.3520
8.3475
8.2264
8.2239
8.2194
8.2067
8.2042
8.2023
8.1996
8.1460
8.1430
8.1392
8.1266
8.1226
8.1198
7.9317
7.9109
7.8342
7.8145
7.7949
7.7506
7.7289
7.5417
7.5263
7.5111
7.4823
7.4607
4.2486
4.2335
2.0919
1.4090
NHAc
S
O
N
N
BocHN
0.94
0.98
1.00
2.01
1.05
2.17
0.92
4.12
8.4
8.3
8.2
8.1
8.0
7.9
7.8
7.7
7.6
7.5
ppm
1.06
2.00
3.19
8.61
10.5
10.0
9.5
9.0
8.5
8.0
7.5
7.0
6.5
6.0
5.5
5.0
4.5
4.0
3.5
3.0
2.5
2.0
1.5
1.0
0.5
ppm

1b

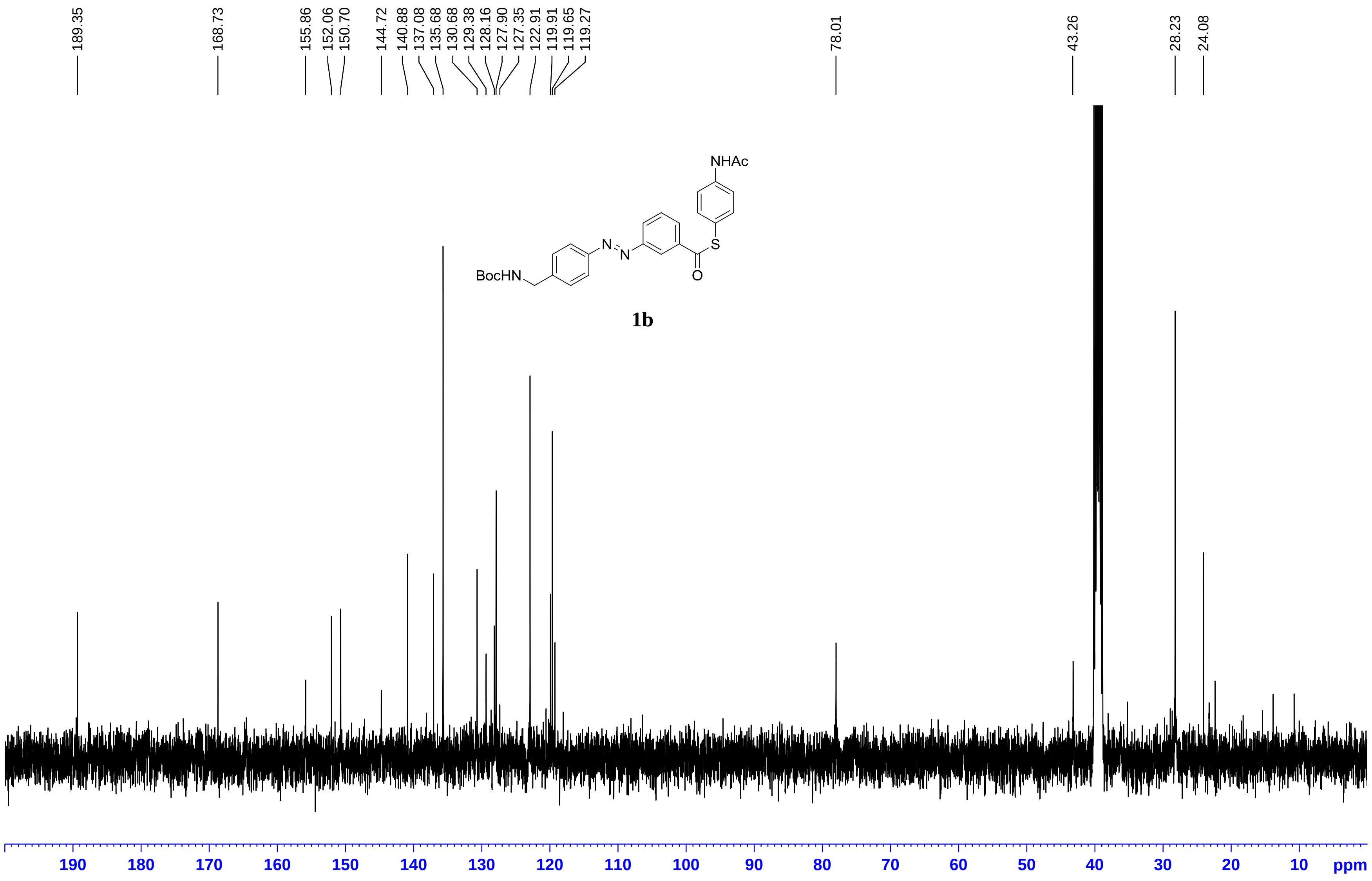
189.35
168.73
155.86
152.06
150.70
144.72
140.88
137.08
135.68
130.68
129.38
128.16
127.90
127.35
122.91
119.91
119.65
119.27
78.01
43.26
28.23
24.08
NHAc
S
O
N
N
BocHN
190
180
170
160
150
140
130
120
110
100
90
80
70
60
50
40
30
20
10
ppm

1b

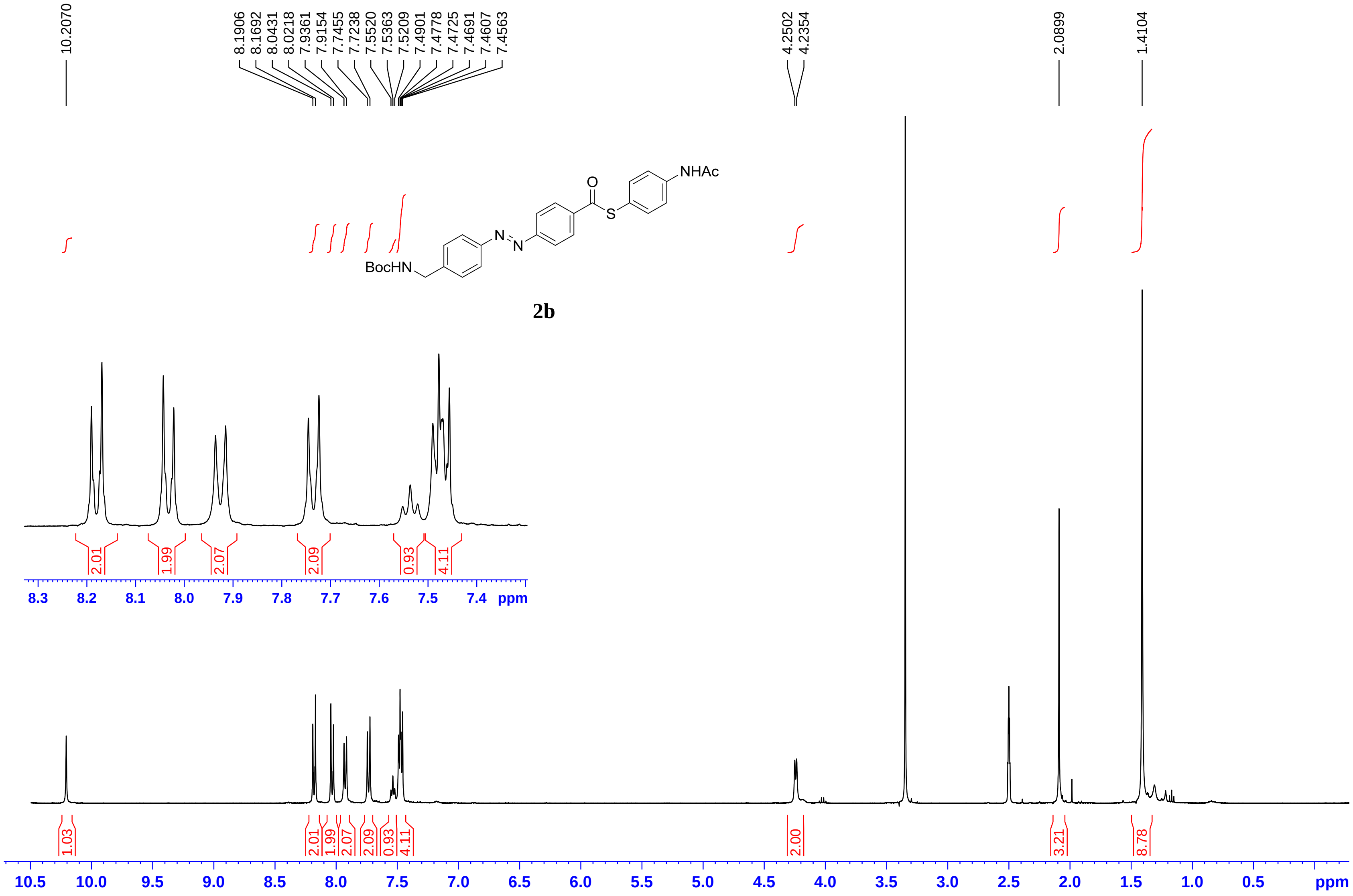
10.2070
8.1906
8.1692
8.0431
8.0218
7.9361
7.9154
7.7455
7.7238
7.5520
7.5363
7.5209
7.4901
7.4778
7.4725
7.4691
7.4607
7.4563
4.2502
4.2354
2.0899
1.4104
NHAc
O
S
N
N
BocHN
2b
2.01
1.99
2.07
2.09
0.93
4.11
8.3 8.2 8.1 8.0 7.9 7.8 7.7 7.6 7.5 7.4 ppm
1.03
2.01
1.99
2.07
2.09
0.93
4.11
2.00
3.21
8.78
10.5 10.0 9.5 9.0 8.5 8.0 7.5 7.0 6.5 6.0 5.5 5.0 4.5 4.0 3.5 3.0 2.5 2.0 1.5 1.0 0.5 ppm

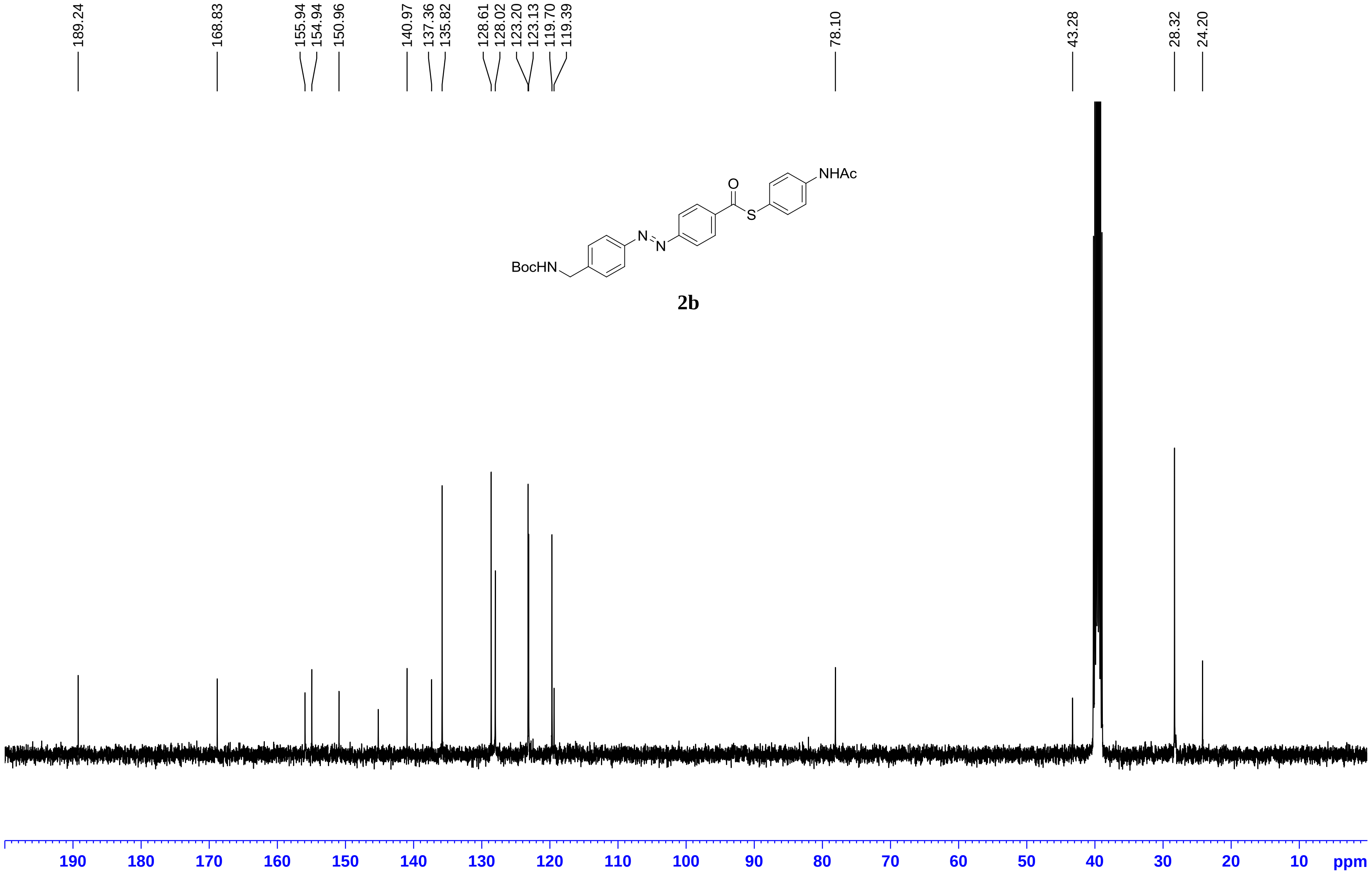
189.24
168.83
155.94
154.94
150.96
140.97
137.36
135.82
128.61
128.02
123.20
123.13
119.70
119.39
78.10
43.28
28.32
24.20
BocHN
N
N
O
S
NHAc
190 180 170 160 150 140 130 120 110 100 90 80 70 60 50 40 30 20 10 ppm

2b

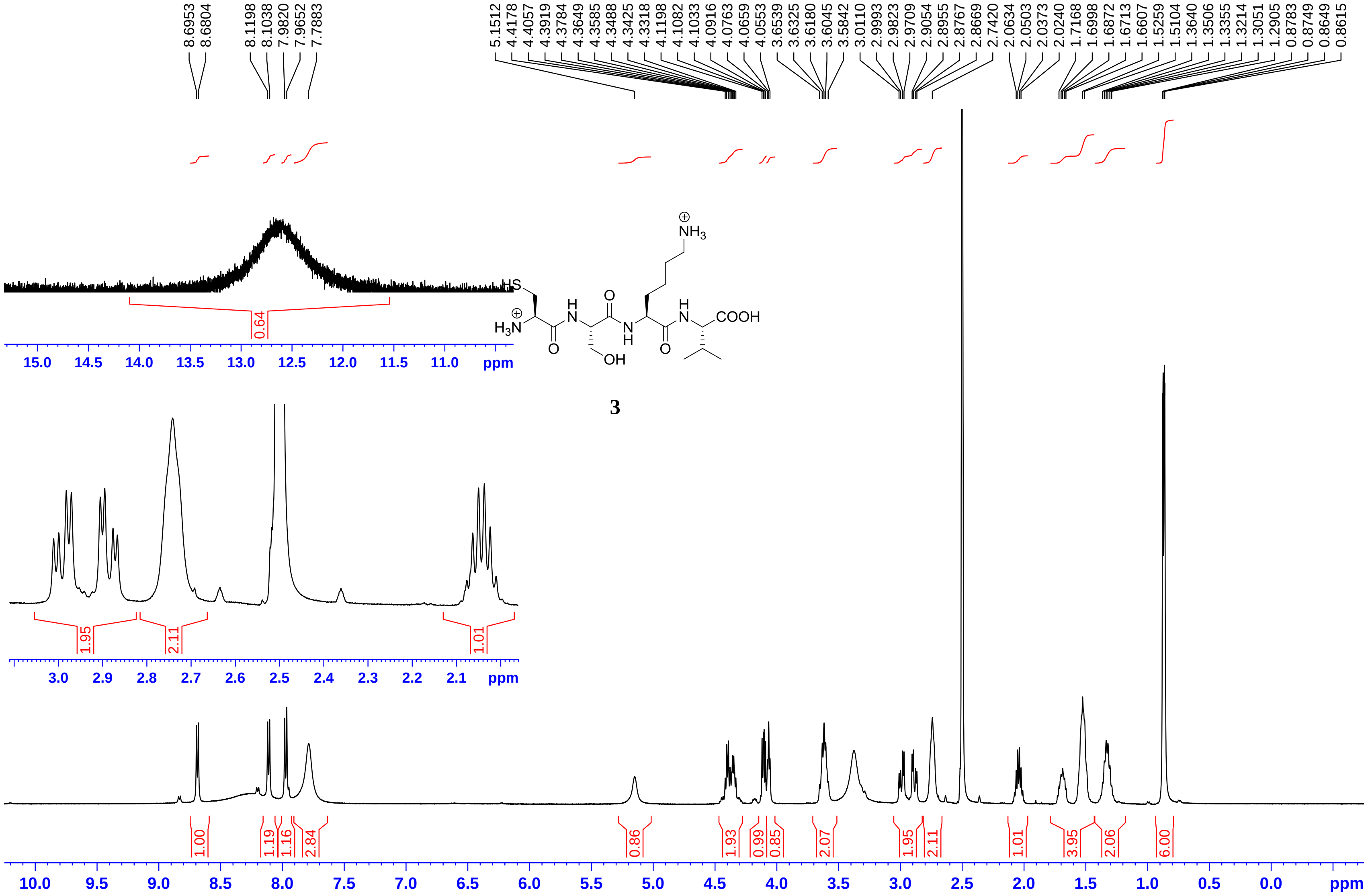
8.6953
8.6804
8.1198
8.1038
7.9820
7.9652
7.7883
5.1512
4.4178
4.4057
4.3919
4.3784
4.3649
4.3585
4.3488
4.3425
4.3318
4.1198
4.1082
4.1033
4.0916
4.0763
4.0659
4.0553
3.6539
3.6325
3.6180
3.6045
3.5842
3.0110
2.9993
2.9823
2.9709
2.9054
2.8955
2.8767
2.8669
2.7420
2.0634
2.0503
2.0373
2.0240
1.7168
1.6998
1.6872
1.6713
1.6607
1.5259
1.5104
1.3640
1.3506
1.3355
1.3214
1.3051
1.2905
0.8783
0.8749
0.8649
0.8615
0.64
ppm
1.95
2.11
1.01
ppm
1.00
1.19
1.16
2.84
0.86
1.93
0.99
0.85
2.07
1.95
2.11
1.01
3.95
2.06
6.00
ppm
HS
H3N
NH3
COOH
OH

3

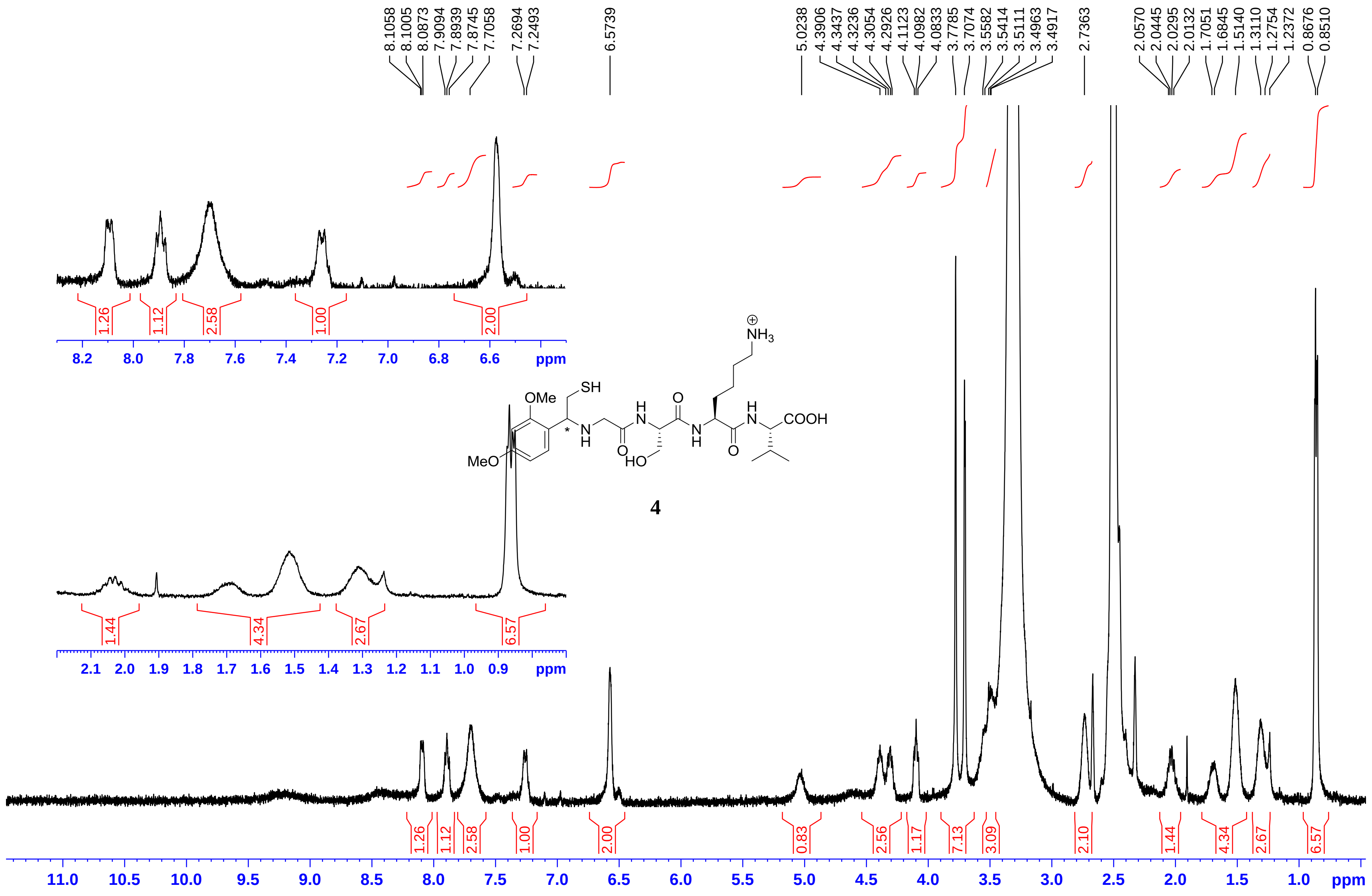
8.1058
8.1005
8.0873
7.9094
7.8939
7.8745
7.7058
7.2694
7.2493
6.5739
5.0238
4.3906
4.3437
4.3236
4.3054
4.2926
4.1123
4.0982
4.0833
3.7785
3.7074
3.5582
3.5414
3.5111
3.4963
3.4917
2.7363
2.0570
2.0445
2.0295
2.0132
1.7051
1.6845
1.5140
1.3110
1.2754
1.2372
0.8676
0.8510
OMe
SH
MeO
NH3
COOH
HO
ppm

4

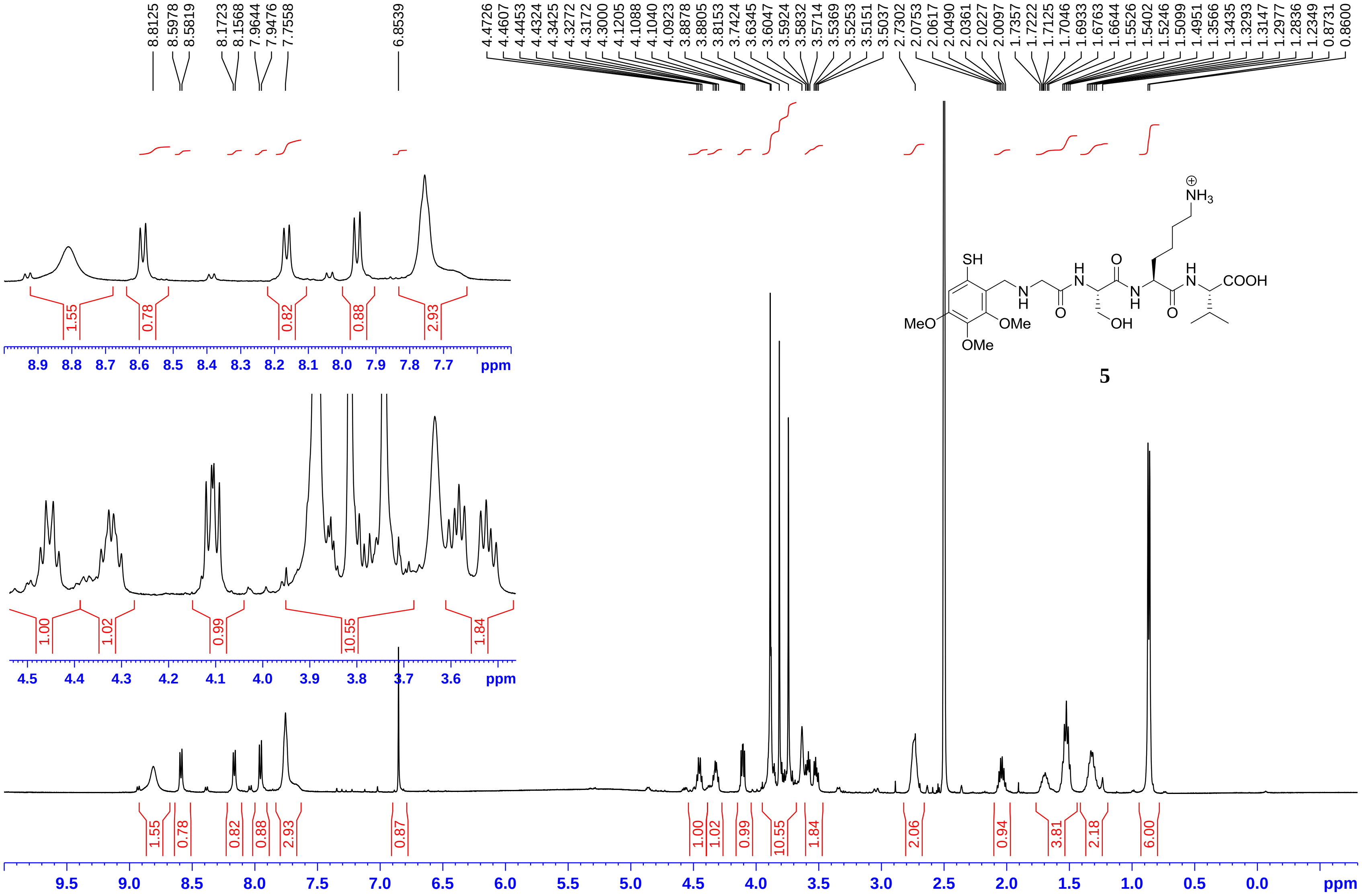
8.8125
8.5978
8.5819
8.1723
8.1568
7.9644
7.9476
7.7558
6.8539
4.4726
4.4607
4.4453
4.4324
4.3425
4.3272
4.3172
4.3000
4.1205
4.1088
4.1040
4.0923
3.8878
3.8805
3.8153
3.7424
3.6345
3.6047
3.5924
3.5832
3.5714
3.5369
3.5253
3.5151
3.5037
2.7302
2.0753
2.0617
2.0490
2.0361
2.0227
2.0097
1.7357
1.7222
1.7125
1.7046
1.6933
1.6763
1.6644
1.5526
1.5402
1.5246
1.5099
1.4951
1.3566
1.3435
1.3293
1.3147
1.2977
1.2836
1.2349
0.8731
0.8600
1.55
0.78
0.82
0.88
2.93
8.9 8.8 8.7 8.6 8.5 8.4 8.3 8.2 8.1 8.0 7.9 7.8 7.7 ppm
1.00
1.02
0.99
10.55
1.84
4.5 4.4 4.3 4.2 4.1 4.0 3.9 3.8 3.7 3.6 ppm
1.55
0.78
0.82
0.88
2.93
0.87
1.00
1.02
0.99
10.55
1.84
2.06
0.94
3.81
2.18
6.00
9.5 9.0 8.5 8.0 7.5 7.0 6.5 6.0 5.5 5.0 4.5 4.0 3.5 3.0 2.5 2.0 1.5 1.0 0.5 0.0 ppm
SH
MeO
OMe
OMe
N
H
O
H
N
O
N
H
OH
NH_3 ⊕
O
H
N
COOH
O
5

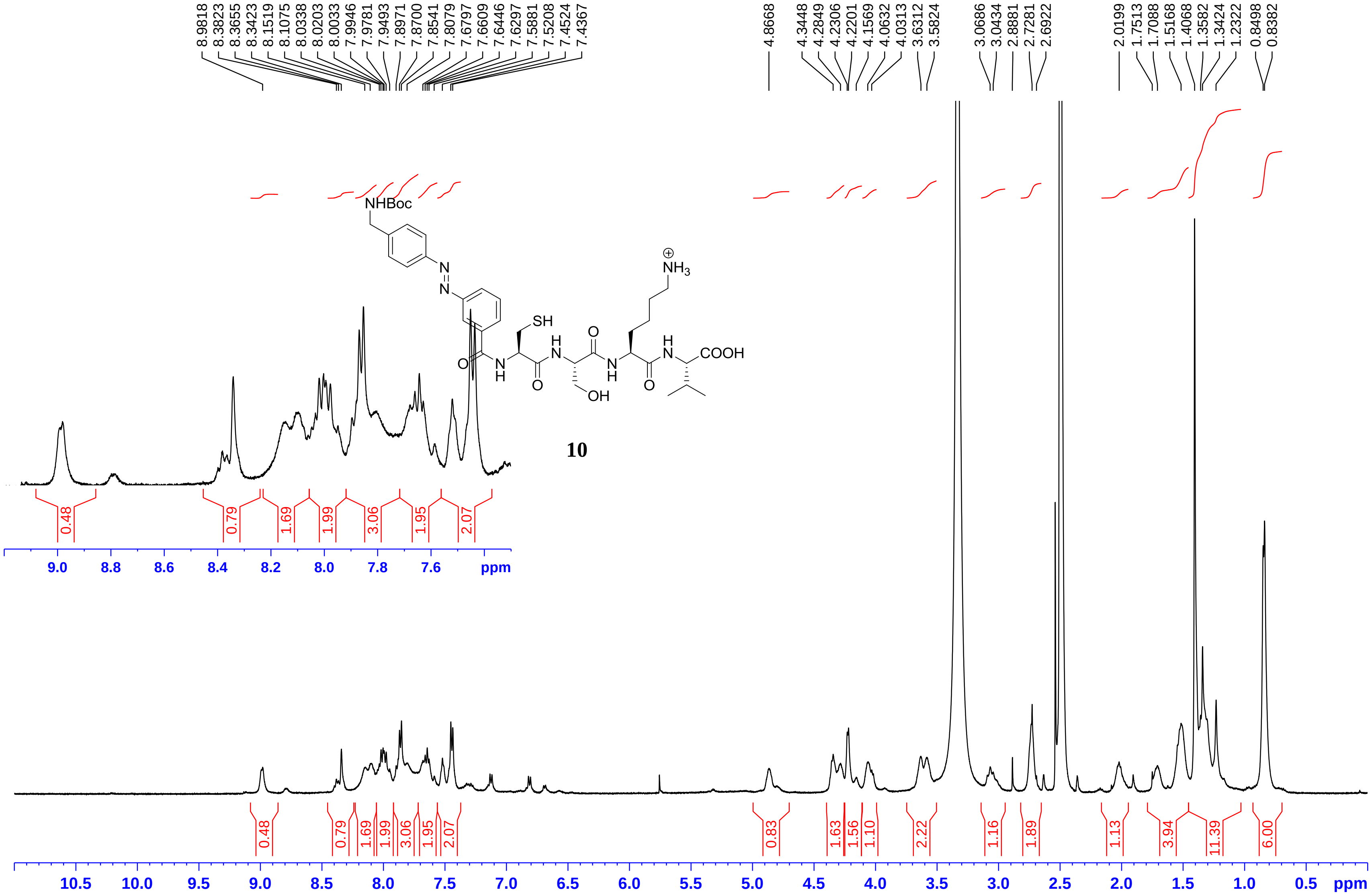

10

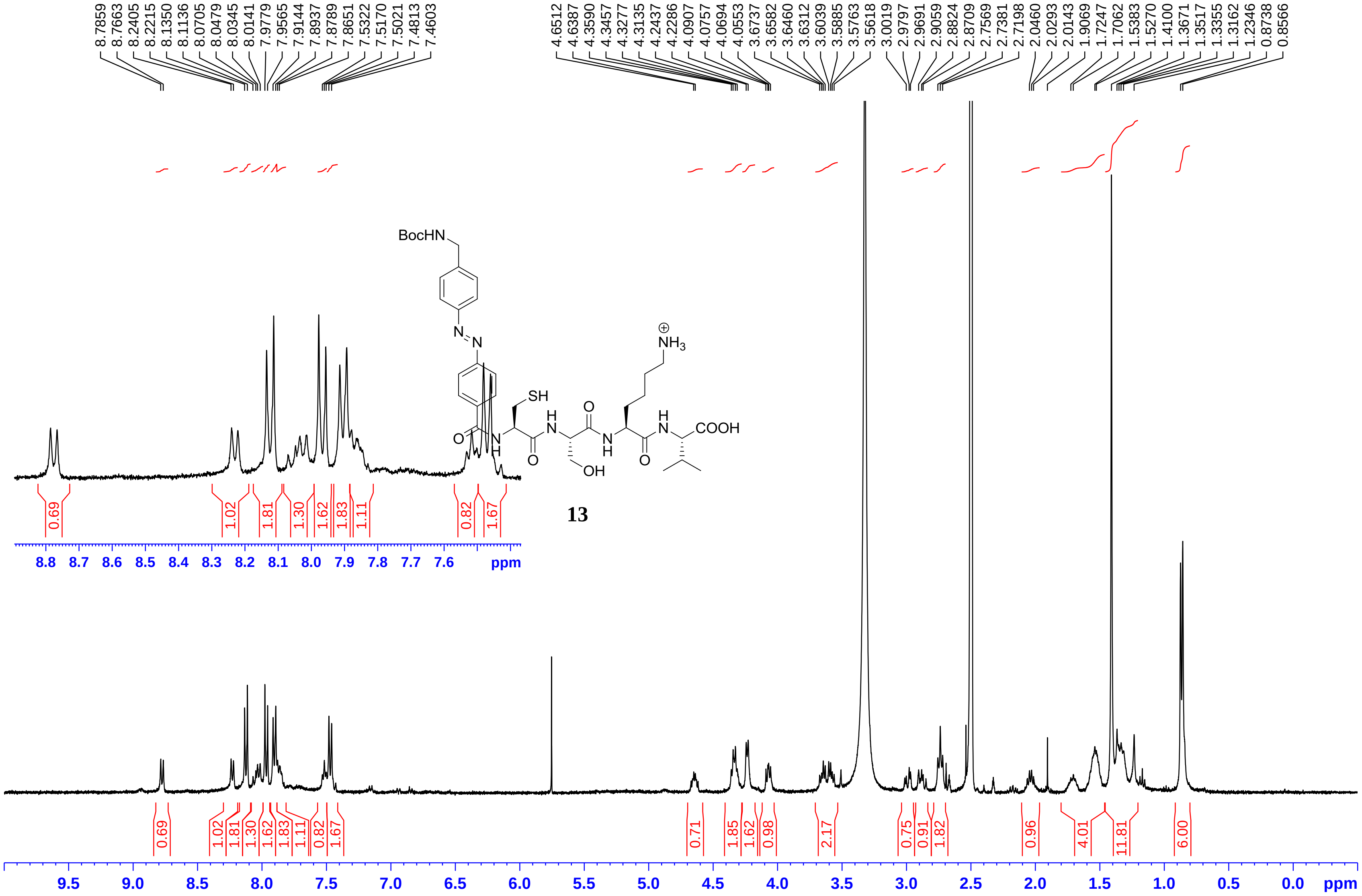
8.7859 8.7663 8.2405 8.2215 8.1350 8.1136 8.0705 8.0479 8.0345 8.0141 7.9779 7.9565 7.9144 7.8937 7.8789 7.8651 7.5322 7.5170 7.5021 7.4813 7.4603
4.6512 4.6387 4.3590 4.3457 4.3277 4.3135 4.2437 4.2286 4.0907 4.0757 4.0694 4.0553 3.6737 3.6582 3.6460 3.6312 3.6039 3.5885 3.5763 3.5618 3.0019 2.9797 2.9691 2.9059 2.8824 2.8709 2.7569 2.7381 2.7198 2.0460 2.0293 2.0143 1.9069 1.7247 1.7062 1.5383 1.5270 1.4100 1.3671 1.3517 1.3355 1.3162 1.2346 0.8738 0.8566
BocHN
N
N
SH
NH3
O
N
H
O
N
H
O
OH
N
H
O
N
H
COOH
13
0.69 1.02 1.81 1.30 1.62 1.83 1.11 0.82 1.67
8.8 8.7 8.6 8.5 8.4 8.3 8.2 8.1 8.0 7.9 7.8 7.7 7.6 ppm
0.69 1.02 1.81 1.30 1.62 1.83 1.11 0.82 1.67 0.71 1.85 1.62 0.98 2.17 0.75 0.91 1.82 0.96 4.01 11.81 6.00
9.5 9.0 8.5 8.0 7.5 7.0 6.5 6.0 5.5 5.0 4.5 4.0 3.5 3.0 2.5 2.0 1.5 1.0 0.5 0.0 ppm

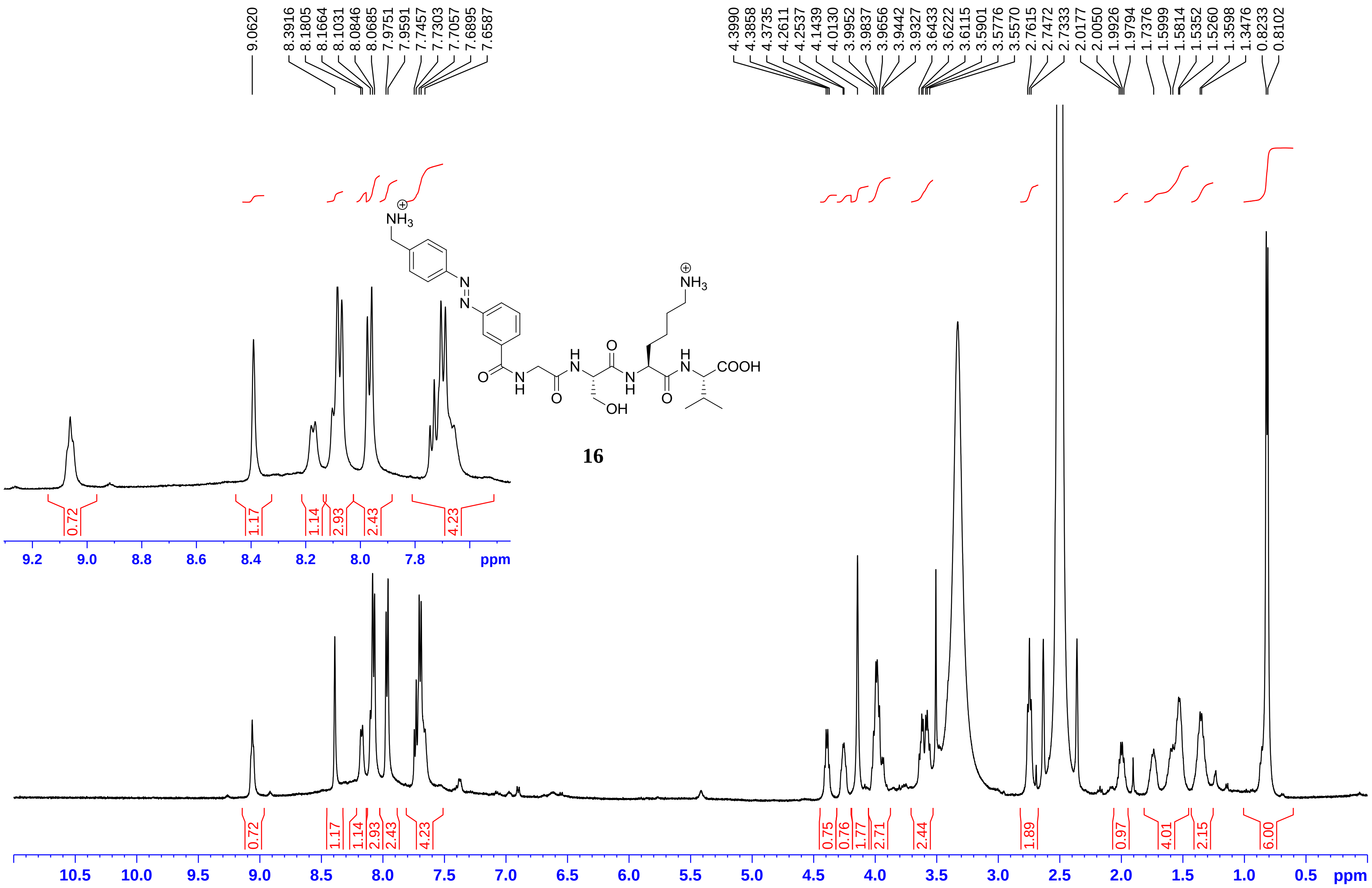
9.0620
8.3916
8.1805
8.1664
8.1031
8.0846
8.0685
7.9751
7.9591
7.7457
7.7303
7.7057
7.6895
7.6587
4.3990
4.3858
4.3735
4.2611
4.2537
4.1439
4.0130
3.9952
3.9837
3.9656
3.9442
3.9327
3.6433
3.6222
3.6115
3.5901
3.5776
3.5570
2.7615
2.7472
2.7333
2.0177
2.0050
1.9926
1.9794
1.7376
1.5999
1.5814
1.5352
1.5260
1.3598
1.3476
0.8233
0.8102
16
0.72
1.17
1.14
2.93
2.43
4.23
0.75
0.76
1.77
2.71
2.44
1.89
0.97
4.01
2.15
6.00
ppm

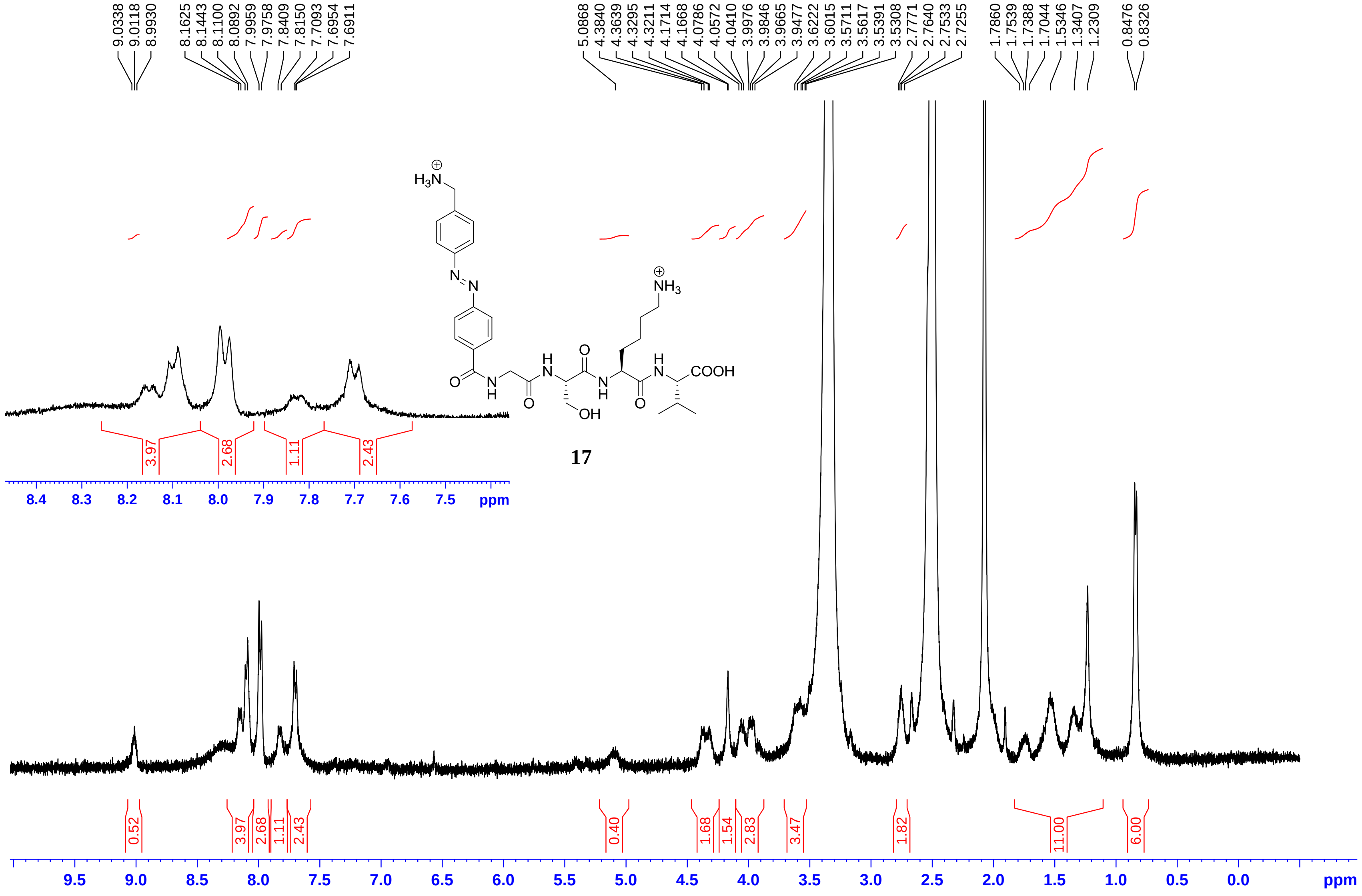
9.0338
9.0118
8.9930
8.1625
8.1443
8.1100
8.0892
7.9959
7.9758
7.8409
7.8150
7.7093
7.6954
7.6911
5.0868
4.3840
4.3639
4.3295
4.3211
4.1714
4.1668
4.0786
4.0572
4.0410
3.9976
3.9846
3.9665
3.9477
3.6222
3.6015
3.5711
3.5617
3.5391
3.5308
2.7771
2.7640
2.7533
2.7255
1.7860
1.7539
1.7388
1.7044
1.5346
1.3407
1.2309
0.8476
0.8326
H3N
N
N
NH3
COOH
OH
17
3.97
2.68
1.11
2.43
8.4 8.3 8.2 8.1 8.0 7.9 7.8 7.7 7.6 7.5 ppm
0.52
3.97
2.68
1.11
2.43
0.40
1.68
1.54
2.83
3.47
1.82
11.00
6.00
9.5 9.0 8.5 8.0 7.5 7.0 6.5 6.0 5.5 5.0 4.5 4.0 3.5 3.0 2.5 2.0 1.5 1.0 0.5 0.0 ppm

References

[1] Priewisch, B.; Rück-Braun, K. *J. Org. Chem.* **2005**, *70*, 2350-2352.

[2] Furlán, R. L. E.; Mata, E. G. *ARKIVOC* **2003**, *10*, 32–40.

[3] von Eggelkraut-Gottanka, R.; Klose, A.; Beck-Sickinger, A. G.; Beyermann, M. *Tetrahedron Lett.* **2003**, *44*, 3551–3554.

[4] Rück-Braun, K.; Kempa, S.; Priewisch, B.; Richter, A.; Seedorff, S.; Wallach, L. *Synthesis* **2009**, *24*, 4256-4267.

[5]

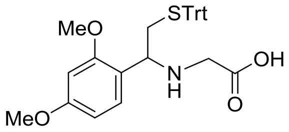

8

2-Glycine-2-(2,4-dimethoxyphenyl) (S-trityl) ethanethiol **8** was prepared by following a literature protocol: Macmillan, D.; Anderson, D. W. *Org. Lett.* **2004**, *6*, 4659-4662.

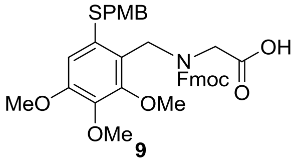

9

3,4,5-Trimethoxyphenyl-(4-methoxybenzyl)-thioether was synthesized from 3,4,5-trimethoxythiophenol and *p*-methoxybenzylchloride according to a procedure published by Dawson et al. (Burlina, F.; Dixson, D. D.; Doyle, R. P.; Chassaing, G.; Boddy, C. N.; Dawson, P. E.; Offer, J. *Chem. Commun.* **2008**, 2785-2787), and 3,4,5-trimethoxythiophenol was prepared from commercially available 3,4,5-trimethoxyaniline (Offer, J.; Boddy, C. N. C.; Dawson, P. E. *J. Am. Chem. Soc.* **2002**, *124*, 4642-4646).

[6] Wu, B.; Chen, J.; Warren, J. D.; Chen, G.; Hua, Z.; Danishefsky, S. J. *Angew. Chem. Int. Ed.* **2006**, *45*, 4116-4125.

[7] Rau, H. In *Photoreactive Organic Thin Films*; Sekkat, Z.; Knoll, W., Eds.; Academic Press: San Diego, **2002**, 3.
